# Hazard assessment of nanomaterials: how to meet the requirements for (next generation) risk assessment

**DOI:** 10.1186/s12989-024-00615-4

**Published:** 2024-12-27

**Authors:** Eleonora Marta Longhin, Ivan Rios-Mondragon, Espen Mariussen, Congying Zheng, Martí Busquets, Agnieszka Gajewicz-Skretna, Ole-Bendik Hofshagen, Neus Gómez Bastus, Victor Franco Puntes, Mihaela Roxana Cimpan, Sergey Shaposhnikov, Maria Dusinska, Elise Rundén-Pran

**Affiliations:** 1 Health Effects Laboratory, Department of Environmental Chemistry and Health Effects, NILU, 2007 Kjeller, Norway; 2 Department of Clinical Dentistry, Faculty of Medicine, University of Bergen, Årstadveien 19, 5009 Bergen, Norway; 3 Norgenotech AS, Ullernchausseén 64, 0379 Oslo, Norway; 4 Oslo Cancer Cluster, Ullernchausseén 64, 0379 Oslo, Norway; 5 Applied Nanoparticles SL, Alaba 88, 08018 Barcelona, Spain; 6 Laboratory of Environmental Chemoinformatics, Faculty of Chemistry, University of Gdansk, Wita Stwosza 63, 80-308 Gdansk, Poland; 7 Institut Català de Nanociència i Nanotecnologia (ICN2), CSIC, The Barcelona Institute of Science and Technology (BIST), Campus UAB, Bellaterra, 08193 Barcelona, Spain; 8 CIBER en Bioingeniería, Biomateriales y Nanomedicina, CIBER-BBN, Spain Institució, 28029 Madrid, Spain; 9 Institució Catalana de Recerca i Estudis Avançats (ICREA), 08010 Barcelona, Spain; 10 Vall d’Hebron Institut de Recerca (VHIR), 08035 Barcelona, Spain; 11 Present Address: Department of Air Quality and Noise, Norwegian Institute of Public Health, Lovisenberggata 8, 0456 Oslo, Norway

**Keywords:** Hazard assessment, Next generation risk assessment, Nanomaterials, New approach methodologies, Safe and sustainable by design

## Abstract

**Background:**

Hazard and risk assessment of nanomaterials (NMs) face challenges due to, among others, the numerous existing nanoforms, discordant data and conflicting results found in the literature, and specific challenges in the application of strategies such as grouping and read-across, emphasizing the need for New Approach Methodologies (NAMs) to support Next Generation Risk Assessment (NGRA). Here these challenges are addressed in a study that couples physico-chemical characterization with in vitro investigations and in silico similarity analyses for nine nanoforms, having different chemical composition, sizes, aggregation states and shapes. For cytotoxicity assessment, three methods (Alamar Blue, Colony Forming Efficiency, and Electric Cell-Substrate Impedance Sensing) are applied in a cross-validation approach to support NAMs implementation into NGRA.

**Results:**

The results highlight the role of physico-chemical properties in eliciting biological responses. Uptake studies reveal distinct cellular morphological changes. The cytotoxicity assessment shows varying responses among NMs, consistent among the three methods used, while only one nanoform gave a positive response in the genotoxicity assessment performed by comet assay.

**Conclusions:**

The study highlights the potential of in silico models to effectively identify biologically active nanoforms based on their physico-chemical properties, reinforcing previous knowledge on the relevance of certain properties, such as aspect ratio. The potential of implementing in vitro methods into NGRA is underlined, cross-validating three cytotoxicity assessment methods, and showcasing their strength in terms of sensitivity and suitability for the testing of NMs.

**Graphical abstract:**

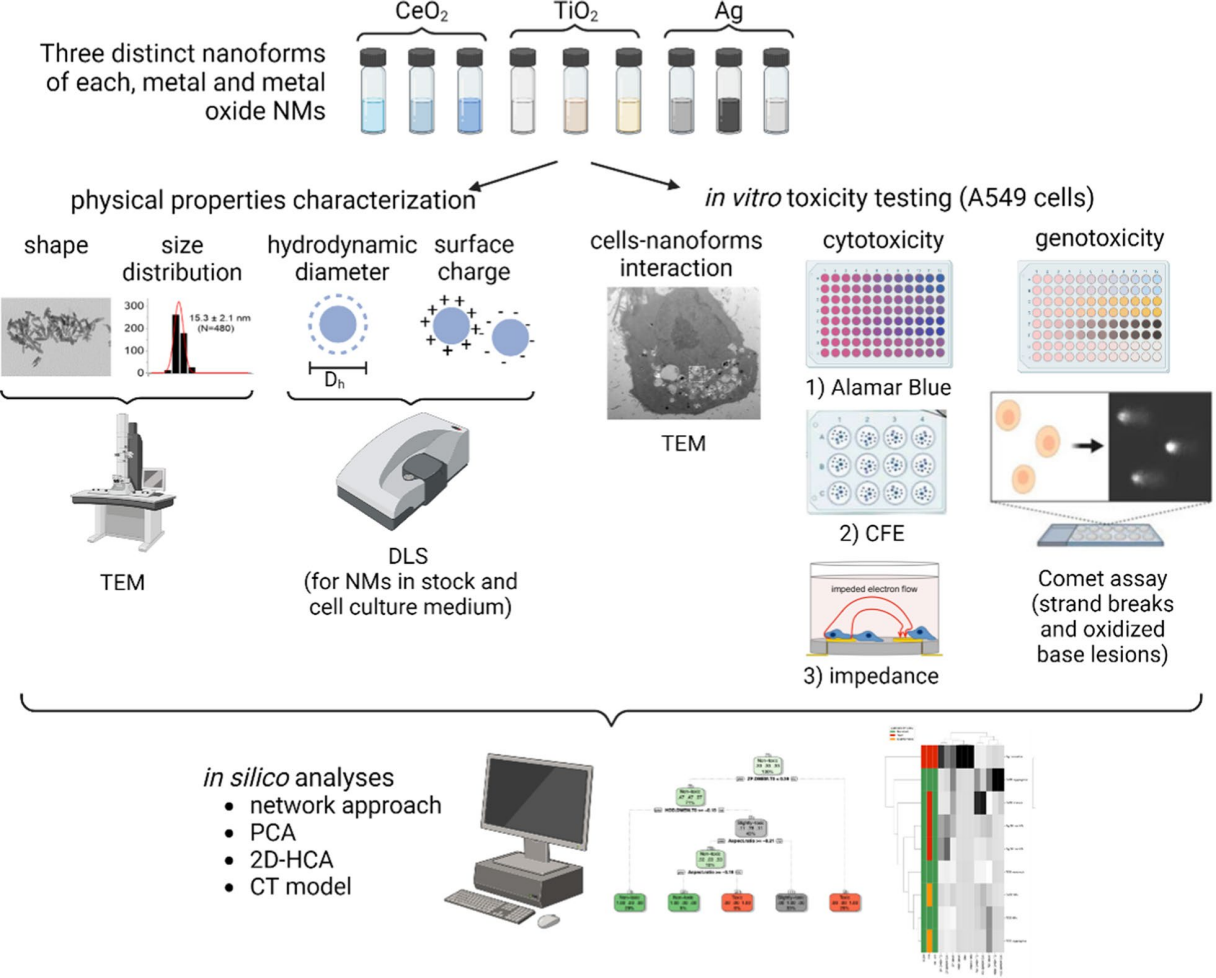
 Created with BioRender.com (publication license obtained)

**Supplementary Information:**

The online version contains supplementary material available at 10.1186/s12989-024-00615-4.

## Background

The quest for improved or specific properties of materials and products such as cosmetics, electronics, and medical applications, has led to an increased production of nanomaterials (NMs) with diverse characteristics. Concern about their safety has been risen, and several NMs have been reported to induce toxic responses in vitro and in vivo [47], including mortality, hepatotoxicity and neurotoxicity [5, 36]. The toxicity mechanisms associated to these adverse outcomes include reactive oxygen species (ROS) formation, DNA damage, inflammation, and disruption of membrane integrity, among others [47]. The number of epidemiological studies is still limited and although findings suggest possible adverse health effects in humans exposed to some NMs, a generalization to all NMs is not appropriate [54]. All these aspects highlight the importance of a sound assessment of the safety of NMs.

Risk assessment (RA) of NMs encounters specific challenges that are not always taken into consideration in hazard assessment investigations. As a result, toxicological studies can face the exclusion from RA evaluations, due to the high risk of bias. Discordant hazard assessment data can be found about the toxicity of NMs, for instance, regarding CeO_2_ NMs’ anti-inflammatory effects and ROS-chelating properties [11, 38], and TiO_2_ NMs’ genotoxicity [16, 35, 46, 63]. Discrepancies may partly stem from the existence of various nanoforms, which even in the presence of the same chemical composition, can exhibit specific toxicological behaviors due to variations in physical properties such as size, shape, surface charge, aggregation/agglomeration state, aspect ratio, and others [27, 26]. Providing enough information about the physico-chemical properties of the materials tested is thus essential to properly distinguish different nanoforms. To address this aspect, the GUIDEnano approach sets quality criteria for toxicological studies, implementing specific parameters for the characterization of NMs [27].

Additionally, considering NMs-related features when performing toxicological studies is crucial to prevent bias, ensure data quality, and thus avoid the exclusion of hazard assessment data from RA. Nano-specific considerations for in vitro studies include the assessment of the cell-NMs interaction/cellular uptake (particularly in case of negative results) [20, 69], and of the possible interference of NMs with the test method applied [23].

Lastly, the use of standardized experimental conditions and dispersion methods for NMs suspensions is needed to obtain reliable and reproducible toxicological results. As the lack of standards and test guidelines (TGs), e.g. OECD TGs, specific for NMs had been recognized as a shortcoming, many efforts towards standard operating procedures (SOPs) harmonization have been done in the recent years through activities such as the Malta initiative, and H2020 projects including PATROLS, RiskGONE, NanoHarmony, and others [17].

In this context, New Approach Methodologies (NAMs), namely in vitro and in silico methods to reduce animal testing and enhance assessment accuracy, are gaining prominence in the transition to next generation RA (NGRA) [56], and in the context of Safe and Sustainable by Design (SSbD) approach. SSbD, aiming at supporting the innovation process of chemicals and materials and at the same time minimizing the health and environment impact [9], is a voluntary approach and a non-legally binding instrument. As such, the implementation of NAMs into this framework occurs without constraints. Although the use of NAMs in the regulatory context requires more consideration, they are already the basis of RA within the Scientific Committee on Consumer Safety (SCCS), due to the EU animal testing ban for cosmetics [55, 65]. The further implementation of NAMs into RA depends on the perception of the reliability and relevance of these methods [48].

Grouping and read-across are strategies implemented in REACH and other legislations to facilitate RA through the categorization of chemicals or substances into groups based on similarities in parameters including molecular structure, physico-chemical properties, toxicological properties. The applicability of these approaches to NMs presents unique challenges due to their complex physico-chemical properties and behavior [19, 20, 26, 42, 49, 60].

Within this context, here we address the challenges faced in the evaluation of NMs’ safety from a NGRA perspective, coupling physico-chemical characterization with in vitro hazard assessment of NMs and in silico similarity analyses. Using the same experimental settings, three different colloids are compared for each of the widely used metal and metal oxides NMs, CeO_2_, TiO_2_, and Ag, for a total of nine different nanoforms. To address the role of size and aggregation status, both highly dispersed and controlled aggregated samples were included in the study, and the NMs colloidal state was characterized in the stock and cell exposure media. For hazard assessment we focused on cyto- and genotoxicity, applying in vitro methods for which standardized methods (such as OECD TGs) are still not available, to support the ongoing efforts towards harmonization, standardization and NAMs development. To ensure the quality of the study and suitability for RA purposes, we implemented recommendations from recent knowledge and requirements for RA of chemicals and NMs, including the GUIDEnano criteria, analyses of the cell-NMs interaction/cellular uptake, and reporting of negative/positive historical controls, and interference controls. Finally, the results were compared by applying similarity analyses using network modeling techniques. This study highlights in a simple and direct way the role of the NMs’ physico-chemical properties in eliciting biological responses, and the potential of in silico analyses in combination with in vitro hazard characterization in supporting NGRA of NMs.

## Materials and methods

### Nanomaterials (NMs)

NMs of the metal oxides CeO_2_, TiO_2_ (anatase), and Ag were provided by Applied Nanoparticles SL (Barcelona, Spain) in stable (colloidal) dispersion in aqueous media. For each chemical composition, three different morphologies and/or colloidal states were used to study a broader and more representative array of commercially available NMs (or nanoforms) as here described:

(i) Dispersions containing single suspended (or highly dispersed) nanoparticles (NPs) i.e., TiO_2_ NPs, CeO_2_ NPs, and Ag NPs (two different sizes–20 and 50 nm).
(ii) Ad hoc and well-controlled mildly aggregated versions of the CeO_2_ and TiO_2_ NPs, i.e., CeO_2_ aggregates and TiO_2_ aggregates, were included to investigate the role of the aggregation status on the NMs’ toxicity.
(iii) NMs with specific shapes, i.e., CeO_2_ stamps, TiO_2_ nanorods, and Ag nanowires were included to address the role of morphology in the NMs’ toxicological behavior.

The CeO_2_ NPs (nominal size 3.5 nm) were synthesized following a basic co-precipitation approach using tetramethylammonium hydroxide (TMAOH) as base in the presence of sodium citrate, based on the procedure described in Ernst et al. [25]. The CeO_2_ aggregates were prepared following a similar basic co-precipitation approach using TMAOH as base, following the procedure described in Cafun et al. [8]. CeO_2_ stamps were produced by basic co-precipitation using hexamethylenetetramine (HMT) based on the procedure described in García et al. [29]. Both TiO_2_ NPs and aggregates colloids were obtained following basic co-precipitation procedures based on Pottier et al. [50], while the TiO_2_ nanorods were produced via a procedure inspired by the findings and synthetic approaches reported by Chang et al. [13]. The 20 nm and 50 nm spheric Ag NPs colloids were produced following a seeded-growth approach based on the method described in Bastús et al. [7]. Finally, Ag nanowires were obtained following a so-called polyol process synthetic approach based on the reduction of metal precursors (in this case silver salts) in hot ethylene glycol in the presence of polyvinylpyrrolidone (PVP) of a given molecular weight and platinum seeds, a modification of the process initially reported by Sun and Xia [61]. All the colloids were purified twice by centrifugation to remove any unwanted unreacted precursors.

The nominal sizes, dispersion medium, stock concentrations and description of the NMs tested are as reported in Table 1.

**Table 1:** List of the NMs used in the study, their nominal size, dispersion medium, stock concentration as reported by the producer. Stock concentration was determined by ICP-MS

Nanoform	Nominal size (nm)	Dispersion medium	Stock concentration by ICP-MS (mg/ml)
CeO_2_ NPs	3.5	10 mM TMAOH	1.75
CeO_2_ aggregates	50	10 mM TMAOH	2.65
CeO_2_ stamps	10 × 10	10 mM TMAOH	2.59
TiO_2_ NPs	8	10 mM TMAOH	2.46
TiO_2_ aggregates	50	10 mM TMAOH	2.38
TiO_2_ nanorods	140 × 40	10 mM TMAOH	2.56
Ag 20 nm NPs	20	5 mM SC + 1 mg/ml 10 kDa PVP	0.84
Ag 50 nm NPs	50	5 mM SC + 1 mg/ml 10 kDa PVP	1.80
Ag nanowires	5000–10,000	5 mM SC + 1 mg/ml 10 kDa PVP	0.94

*TMAOH* Tetramethylammonium hydroxide; *SC* sodium citrate; *PVP* polyvinylpirrolidone

The NMs were provided in stable dispersions (colloids) and stored at 4 °C in the dark. For further testing they were vigorously hand-shaken before use to ensure homogeneous sampling, as recommended by the producer. No further treatment e.g., sonication or addition of serum to aid stability was used. The stability throughout the duration of the study was confirmed by the physico-chemical characterization analyses, particularly the UV–vis and DLS investigations, reported below.

### NMs physico-chemical characterization

The studied materials were extensively characterized for their physico-chemical properties, including morphology, size, colloidal state, and surface electrical charge, both in their pristine status (stock preparations) and in the preparations for the toxicological analyses (dispersions in cell culture medium), as described below.

### Size distribution and shape

**Fig. 1:**
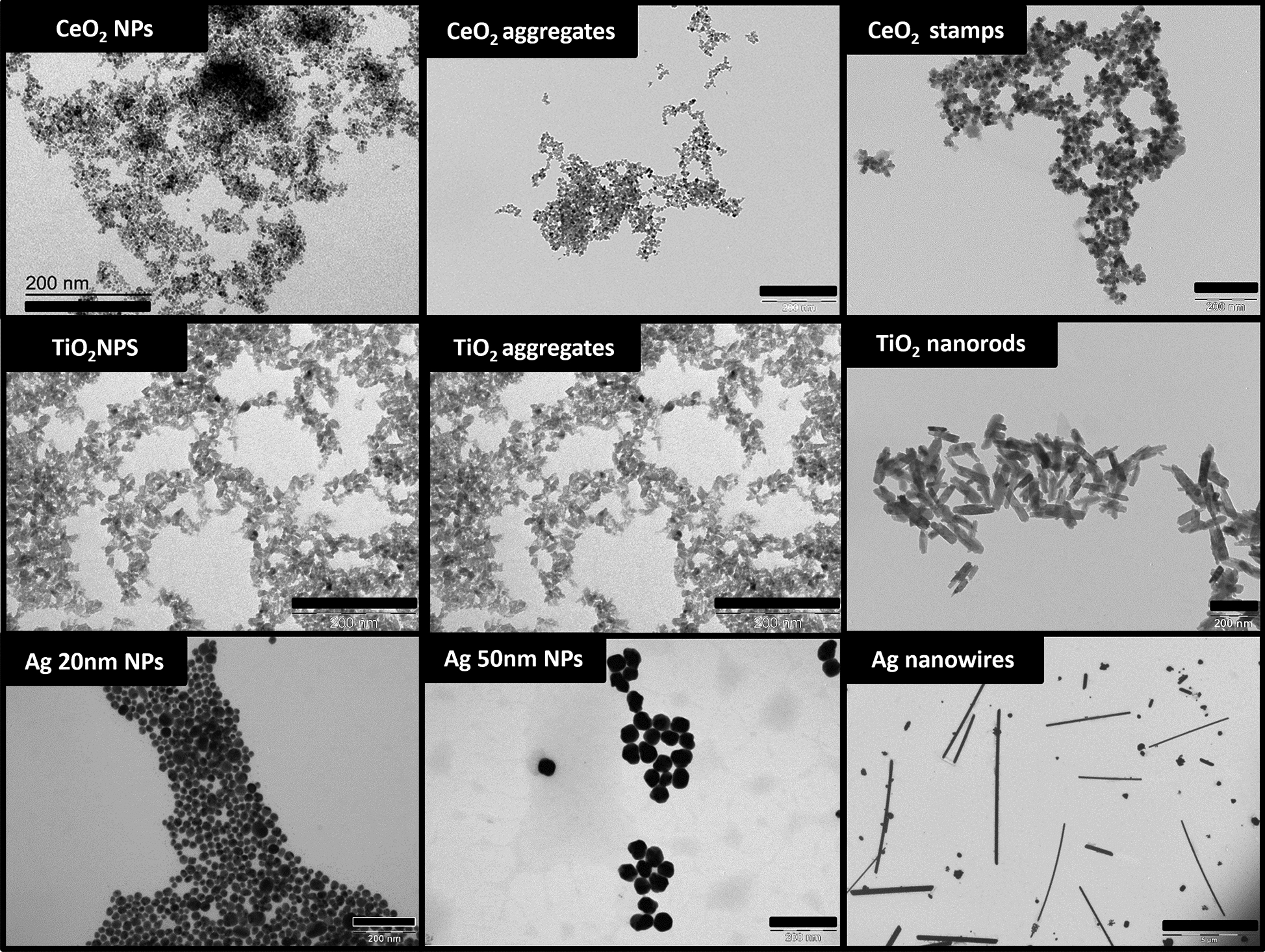
Representative TEM images of the NMs in stock solutions. Magnification bar (black bar) measuring 200 nm for all NMs TEM images except for Ag Nanowires (5000 nm)

For the NMs characterization of pristine size distribution and shape, images were acquired using a JEOL1010 transmission electron microscope (TEM; JEOL, Japan) working at 80 keV. For the samples’ preparation, formvar-coated and carbon-stabilized 200-mesh copper grids (Ted-pella Inc., USA) were dipped in aliquots of the nine NMs colloids with dilutions ranging from 1:1 to 1:10 in milliQ water and left to dry for at least 12 h. ImageJ software (NIH, USA) was used to process the acquired TEM images to calculate mean size and size distribution of the single particles in the NMs preparations. For the anisotropic NMs (e.g. Ag nanowires, TiO_2_ nanorods, etc.), both longitudinal and transversal (length and width) dimensions were assessed by TEM to better define their morphology. Mean size and size distribution were calculated for each NM, using a minimum of 480 counts and a maximum of 5160 counts (as reported in supplementary material S1 Fig. 1) to obtain sufficient statistical significance.

Based on the TEM observations and size measurements, the shape (morphology) of the NMs was also described, and the NMs’ aspect ratio was calculated as the highest to the lowest dimension.

### Hydrodynamic diameter (HDD) and surface charge

The hydrodynamic diameter (HDD), the polydispersity index (PDI), and the surface charge of the pristine NMs (stock preparations) were measured by Dynamic Light Scattering (DLS), and Zeta Potential (ζ-Potential) on a Malvern Zetasizer Nano ZS90 which incorporates a Zeta potential analyser (Malvern Instruments Ltd, Worcestershire, UK). In order to be within the technical experimental limits, samples were diluted in the range 1:10–1:200 in milliQ water depending on the NMs concentration and optical properties. In addition, DLS and ζ-Potential measurements were performed on the particles dispersed in cell culture medium (DMEM supplemented with 10% fetal bovine serum (FBS)), at the beginning (0 h) and at the end (24 h) of cell exposure, to assess the stability of the colloids along the study duration. For these measurements, the particles were dispersed at 100 µg/ml, which was the highest concentration tested for cell exposure. Three independent measurements were performed for each condition and the results are presented as mean ± standard deviation.

### UV–visible absorption

The analysis of UV–Visible absorption spectra of the NMs colloids provides information on the stability of the colloids and was performed using an Agilent Cary 60 UV–Vis Spectrophotometer, setting spectra measuring limits between 300 and 800 nm. Dilution factors were applied to each NM colloid depending on the intensity of their bandgap or surface plasmon resonance peak (SPR), to prevent absorbance saturation. In detail, these dilution factors were 1:25 for CeO_2_ NPs, 1:40 for CeO_2_ aggregates, 1:10 for CeO_2_ stamps, 1:200 for all the three TiO_2_ NMs, 1:125 for Ag 20 nm NPs and 1:50 for both Ag 50 nm NPs and Ag nanowires.

### X-ray photoelectron spectroscopy

X-ray Photoelectron Spectroscopy (XPS) of CeO_2_ NPs and CeO_2_ stamps was employed to determine Ce3 + /Ce4 + species, recording the core level XPS spectra of Ce 3d. The XPS analysis was performed on a SPECS system equipped with a monochromatic Al source operating at 300 W and a Phoibos 150 analyzer. The pass energy of the hemispherical analyzer was set at 20 eV, and the energy step of high-resolution spectra was set at 0.05 eV. Binding energy (BE) values were referred to the C 1 s peak at 285.0 eV. Data processing was performed with the CasaXPS software. Cerium 3d spectra were analyzed using six peaks for Ce4 + (V, V″, V‴, U, U″ and U‴), corresponding to three pairs of spin–orbit doublets, and four peaks (two doublets) for Ce3 + (V0, V′, U0 and U′), based on the peak positions reported by [45], where U and V refer to the 3d3/2 and 3d5/2 spin–orbit components, respectively. Samples were prepared by drop-casting the sample onto a clean silicon wafer. The results are reported in Supplementary material 2.

### Cell culturing

The human lung epithelial cell line A549 (ATCC, Manassas, VA, USA; isolated from the lung of a 58-year-old, white male with carcinoma; passage number below 15) was maintained in DMEM medium supplemented with 10% FBS (product no. 26140079, Thermo Fisher Scientific) and 1% penicillin/streptomycin (Pen-Strep, product no. 15070063, Thermo Fisher Scientific), in an incubator with humidified atmosphere at 37 °C, 5% CO_2_. The cells were cultured at a density of approximately 1.3 × 10^4^ cells/cm^2^ in vented cell culture flasks and sub-cultured twice per week by dry trypsinization (0.25% trypsin for 2–4 min at 37 °C). The cells were seeded for experiments and exposed to NMs as described below.

### Cell-NMs interaction and uptake

Intracellular uptake was assessed by TEM imaging of cells exposed to the NMs. The A549 cells were seeded at a density of 25 000 cells/cm^2^ in 6-well plates and incubated at 37 °C and 5% CO_2_ for 24 h. Then, the cells were exposed to the different NMs at 50 μg/ml and further cultured for 24 h. Afterwards, the cells were fixed with 2.5% glutaraldehyde/2% paraformaldehyde in 0.1 M Na-cacodylate at 4 °C for 24 h. Fixed samples were then washed and postfixed with 1% osmium tetroxide in Na-cacodylate at 4 °C for 60 min. After washing, the samples were dehydrated in ethanol series (30–100%), embedded in Agar 100 resin (Agar Scientific Ltd., UK) with help of gelatine capsules (Agar Scientific Ltd., UK) to obtain blocks with cells released from the plastic plate. The embedded samples were sectioned, mounted on TEM grids and imaged (JEOL-JEM-1230) at 60 kV.

### Cytotoxicity

Cytotoxicity can be assessed by methods based on diverse principles and cell functions. From a regulatory perspective, the verification of the results using at least two independent in vitro assays per endpoint is recommended [20]. Here we used the Alamar Blue (AB) assay, which assesses the metabolic activity of cells, the colony forming efficiency assay (CFE) assay, based on the ability of cells to survive and form colonies, and the electric cell-substrate impedance sensing (ECSIS), based on cell coverage of the electrodes surface and cell membrane integrity. Whenever possible, the EC_50_ was calculated as described in the statistical analyses section. The NMs were categorized as non-toxic, slightly toxic, or toxic through the scoring system described in El Yamani et al. [21]. Briefly, the system categorizes the NMs based on the cytotoxicity value reached at the maximum concentration tested and the value of EC_50_. The results of the three different methods were then compared and used in the similarity analyses as described below.

### Alamar blue (AB)

For the AB assay the cells were seeded in 96-well plates at 1.2 × 10^4^ cells/well. The next day, the cells were exposed to the NMs (concentrations: 10, 20, 50, 80 and 100 μg/ml) for 3 or 24 h. Ag nanowires were cytotoxic at the selected concentrations, thus lower concentrations were added (0.3, 1, 2, 5 μg/ml). Unexposed cells were run in parallel as negative control (NC or 0 μg/ml), while Chlorpromazine (50 µM) was used as positive control (PC). The particles’ dispersion medium (indicated in Table 1) was used as dispersion control (DC) at two different concentrations, corresponding to the highest exposure concentration (DC_H_; 100 μg/ml), and a lower one (DC_L_; 10 μg/ml). Three independent experiments were performed, with samples run in duplicate in each experiment.

At the end of exposure, the cells were incubated for 3 h with fresh culture medium supplemented with 10% AB staining solution, and the fluorescence signal of AB was detected on a microplate reader (FLUOstar OPTIMA, excitation 530 nm, emission 590 nm). Four reading replicates were prepared from each sample for the fluorescence reading, as described in Longhin et al. [41].

After subtracting the blank value (wells with only medium and Alamar Blue solution), the relative cell viability of exposed samples was calculated with respect to NC cells (unexposed samples).

Possible interference of the particles with the assay was investigated by incubating the NMs with AB staining solution alone (no cells) and compared with the blank value. Relative fluorescence intensity < 10% is within the method variability, thus indicating no significant interference is present.

Experiments were considered valid when the exposure to the PC resulted in a significant reduction (at least 50%) of relative fluorescence intensity compared to the NC.

### Colony forming efficiency (CFE)

Long-term cytotoxicity was assessed by the colony forming efficiency (CFE), which is based on the ability of the cells to survive and form colonies. The CFE assay is particularly suitable for the use with NMs since it does not present problems of interference of NMs with the test performance or readout [53].

Approximately 30 cells/well were seeded on 12-well plates and left to settle for 1 h before exposure. Six replicate wells were exposed to each NM concentration (CeO_2_ and TiO_2_ NMs: 1.14, 3.8, 11.4, 38, 114 and 380 μg/ml; Ag NMs: 0.038, 0.114, 0.38, 1.14, 3.8, 11.4 μg/ml), NC (0 μg/ml), DC (DC_H_: 380 or 11.4 μg/ml; DC_L_: 10% of DC_H_), and PC (30 µM chlorpromazine). The cells were incubated for 9–12 days for colonies to be formed, and then stained with 1% methylene blue for 1 h. The staining medium was removed, and the number of colonies counted. The results were normalized to the unexposed control (set to 100% colony forming efficiency).

Experiments were considered valid when the exposure to the PC resulted in a significant reduction (at least 50%) of colonies compared to the NC, according to the acceptance criteria reported in Rundén-Pran et al. [53]. Representative images of the CFE assay for positive and negative controls are reported in Supplementary material 5 (S5 Fig. 1).

### Electric cell-substrate impedance sensing (ECSIS)

The ECSIS was used to assess in a label-free and thus, less prone for interferences manner, the impact of the different NMs on the proliferation and viability of A549 cells [15]. This technique monitors cell viability, cell number, cell-substrate and cell–cell contact in real-time. All these variables affect the current flow across the electrode array onto which cells grow. The changes in the current flow are measured to determine electrical impedance, which is reported by the system as a dimensionless cell index (CI) value. The CI is directly proportional to cell surface coverage and integrity of the cell membrane.

The A549 cells were seeded at a density of 25 000 cells/cm^2^ in 16-well E-plates (Agilent) containing a microelectrode array in the bottom of the wells. Then, E-plates were fitted in the real-time impedance analyser (xCELLigence RTCA, Agilent) and incubated at 37 °C and 5% CO_2_. Electrical impedance was monitored in real-time every 15 min at a 10 kHz AC frequency. After 24 h, the cells were exposed to the different NMs at concentrations of 10, 50 and 100 μg/ml (5, 25 and 50 μg/cm^2^, respectively). Dispersion media controls (DC) were prepared by incubating the cells in the presence of the respective NM’s dispersion media (TMAOH or sodium citrate plus 10 K PVP as indicated in Table 1) at a concentration corresponding to the volume of dispersion media contained in the highest NM concentration tested. In addition, cell-free wells were included to monitor the cell culture medium- and eventual NM-derived impedance background; to note, only the highest concentration of the NMs was used to monitor background. After the addition of NMs, the E-plates were fitted back in the impedance analyser and cells were further cultured for 24 h. For CI analysis, background values were subtracted from the respective exposure conditions for each time-point. These corrected CI(t) values were normalised to the CI value at time-point 24 h, right before the beginning of NM-exposure, to take into account possible differences in number of cells seeded and uniformity of cell distribution onto the electrode array. Finally, the fold-change vs. control was calculated at the end of the 24 h exposure period (48 h total incubation time). All conditions were tested in duplicate wells and the data presented correspond to the mean of at least three independent experiments.

### Genotoxicity

The DNA damage (genotoxicity) was investigated by the single cell gel electrophoresis, or comet assay (CA). DNA strand breaks (SB) were detected by the standard alkaline comet assay, while the modified version of the assay was performed by using the enzyme formamidopyrimidine DNA glycosylase (Fpg, kind gift from NorGenoTec AS, Norway) to specifically detect oxidized base lesions.

The cells were seeded in 96-well plates at a density of 1.2 × 10^4^ cells/well. The next day the cells were exposed to the NMs (concentrations: 10, 20, 50, 80 and 100 μg/ml, plus 0.3, 1, 2, 5 μg/ml for Ag nanowires) for 3 or 24 h. Unexposed cells were run in parallel as NC (0 μg/ml), while methyl methanesulfonate (MMS, 200 µM) was used as PC. As for the AB assay, two DC concentrations were used i.e., 10 (DC_L_) and 100 (DC_H_) μg/ml. For each of these treatments, two technical replica (duplicates) samples were exposed in every experiment, and at least three independent experiments were performed.

After exposure, the cells were detached from the plates by trypsinization, resuspended and embedded in low-melting-point agarose on microscope slides. Parallel slides were prepared for all samples, one slide was used in the standard version of the CA, and one in the Fpg modified assay. All the slides were incubated for 1 h in lysis solution (2.5 M NaCl, 0.1 M EDTA, 10 mM Tris, 10% v/v Triton X-100, pH 10, 4 °C) to dissolve membranes and cytoplasm of the cells and expose the nuclei. The slides for the Fpg modified assay were then washed 10 min twice in buffer F (40 mM HEPES, 0.1 M KCl, 0.5 mM EDTA, 0.2 mg/mL BSA, pH 8, 4 °C), and incubated with the proper dilution of Fpg enzyme, in a humid box at 37 °C for 30 min. All the slides underwent unwinding of the supercoiled DNA by incubation in the electrophoresis solution (0.3 M NaOH, 1 mM EDTA, pH > 13, 4 °C) for 20 min at 4 °C. The electrophoresis was then run in the same buffer for 20 min at 4 °C (25 V, 1.25 V/cm, Consort EV202), to separate the loops of broken DNA strands from the nucleus (comet’s head) into a comet’s tail. Afterwards the slides were washed in PBS and H_2_O for 5 min each and let to dry overnight before staining with SYBR gold (Sigma-Aldrich, USA).

As a control of the correct test performance (lysis and electrophoresis), one slide with embedded cells was exposed to H_2_O_2_ (50 µM, Sigma-Aldrich, Germany) for 5 min before lysis, and the generation of DNA SBs was assessed. As a control of the Fpg activity, the formation of oxidative damage to the DNA was assessed in A549 cells exposed to the photosensitizer Ro 19–8022 (2 µM, kindly provided by Hoffmann La Roche) and light irradiated.

The scoring of the cells’ nuclei (or comets) was performed on a fluorescence microscope (DMI 6000 B, Leica Microsystems, Germany) equipped with a SYBR photographic filter (Thermo Fischer Scientific, USA) and the Comet Assay IV 4.3.1 software (Perceptive Instruments, UK). The investigator was blinded to the group allocation during scoring. The percentage of DNA in tail was taken as measure of the DNA damage. The median of 50 cells per each sample’s duplicate was calculated (100 comets per sample), and the average of the duplicates’ median was reported. The net oxidatively damaged DNA (net Fpg) was calculated as the difference between the Fpg-modified CA (Fpg) and the standard assay (SB), thus these values are here reported as relative percentage of DNA in tail. Data are presented as mean ± SD of at least three independent experiments.

Based on the CA results, NMs were categorized for their genotoxic effect as positive, negative or equivocal according to the scoring system presented in El Yamani et al. [21], which is based on the statistical significance i) of the effect at tested concentrations and ii) of the concentration–response relationship. The categorization results were used in the similarity analyses as described below.

To support data quality, and ensure consistency with previous studies, historical control data are reported in supplementary material S4 Table 5. Experiments fulfilling the acceptance criteria reported in [21, 22] (NC samples with background DNA damage within historical control data, and expected response of the positive controls) were included in the study. Experiments not fulfilling the acceptance criteria were excluded.

**Fig. 2:**
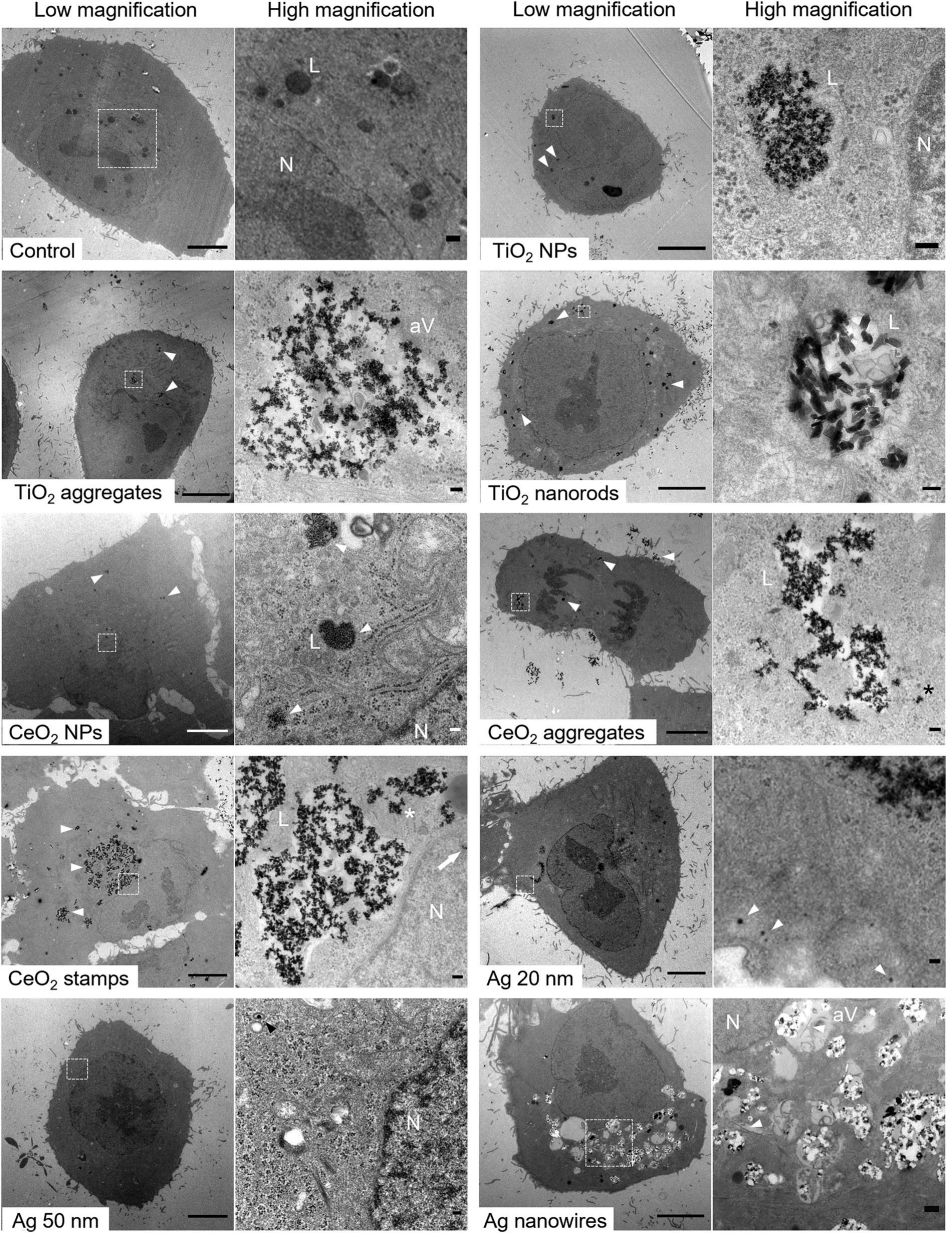
Transmission electron micrographs of A549 cells treated for 24 h with 50 μg/ml of the indicated NMs and untreated control. Low and high magnification images of the selected area (dashed box) are shown. All NMs are readily taken up by the cells and accumulate primarily in membrane-enclosed organelles. Arrowheads indicate examples of NM clusters. Arrow points to CeO_2_ stamps in the nucleus. Asterisks indicate examples of non-membrane-enclosed NMs. N (nucleus), L (lysosomes) and aV (autophagic-like vacuoles). Scalebars: 5 μm in low magnifications, 100 nm in high magnifications and 400 nm in high magnification for control (unexposed cells)

Representative images of the comet assay for NC, DC, PC and cells exposed to NMs are reported in Supplementary material 5 (S5 Fig. 2).

### Similarity analyses

Similarity analysis is a data mining technique used to explore the relationships and patterns among data points (here NMs). Its primary objective is to identify (dis)similarities between pairs of data points, facilitating the grouping of similar ones together and gaining insights into the underlying structures within the data. This study utilizes two well-known unsupervised machine learning techniques for similarity analysis: hierarchical clustering (HCA) and principal component analysis (PCA).

The fundamental concept behind HCA is to cluster similar data points based on the assumption that objects closer to each other in a multi-dimensional space are more alike than those farther apart. In hierarchical cluster analysis, the similarity between data points is measured through distance metrics. Among other distance metrics, the most commonly used are Euclidean distance, Manhattan distance, Minkowski distance, and Pearson correlation coefficient. The distance metric considered in this work was the Euclidean distance, which is calculated as the straight-line distance between two data points in a multi-dimensional space.

The PCA relies on the foundational assumption that the initial principal components, derived as linear combinations of the original features, often reveal the most significant patterns or structures within the data. In essence, these high-variance dimensions capture crucial information about the relationship between variables. Thus, the primary goal of PCA is to determine the directions along which the data exhibits the most variation. Through projecting the original data onto the space of principal components, it becomes possible to identify clusters of similar objects (e.g., NMs) within the data, identify the most influential variables in the original dataset, and investigate the relationship between data points and variables.

All chemometric and in silico analyses were performed using R software (v4.0.3) with the following packages: qgraph [24], factoextra [34], caret [37], ggplot2 [67], smotefamily [58] and rpart [62].

The descriptors used in the similarity analyses included the physico-chemical parameters reported in Tables 2 and 3 and the toxicity parameters reported in Tables 4 and 5. The physico-chemical parameters included NMs’ size (by TEM), HDD (by intensity), surface charge (ζ-Potential, mean value in mV), morphology. Aspect ratio was used as a parameter of morphology, and the first term of the aspect ratio expression (e.g., 1.25 in case of aspect ratio 1.25:1) was used as a variable in the in silico analyses (Table 2). In the case of Ag nanowires, presenting particles of different lengths, a range for aspect ratio was provided (100:1–50:1), and the descriptor for in silico analyses was calculated as an average of this range (75). For the flat CeO_2_ stamps where the three dimensions are described as 3.75:3.75:1, the variable in the in silico analyses was calculated by the average of the three terms, i.e. 2.8.

For cytotoxicity and genotoxicity parameters, a toxicity score and categorization system was applied as described in El Yamani et al. [21] and reported above.

### Statistical analyses

The statistical analyses were performed with the software GraphPad Prism 8.0.1. Ordinary one-way ANOVA with Dunnett’s post-hoc test used to analyse the difference of the samples versus the NC (0 μg/ml). Differences with *p* < 0.05 were considered statistically significant. The half maximal effective concentration (EC_50_) was calculated by nonlinear regression analysis by the four parameters Hill-equation. Linear regression analysis was used to assess the concentration–response relationship. The slope was considered significantly non-zero for *p* < 0.05.

## Results

### NMs physico-chemical characterization

The analysis of the NMs’ physico-chemical size distribution performed in the stock preparations is here reported in Fig. 1, Table 2 and Supplementary material 1. The TEM images in Fig. 1 show the particles’ shape and size in the stock preparations. As the NMs were transferred to the TEM grids by precipitation, or drying, the images are not representative of the aggregation status of the materials in suspension. The size by TEM reported in Table 2 and size distribution graphs reported in Supplementary material 1 thus indicate the size of the single particles in the preparations. Based on these data, the aspect ratio of NMs is calculated as the highest to the lowest dimension. For spheric NMs the aspect ratio is thus 1:1, while spheric NMs showing some degree of anisotropy were given an additional factor of 0.25 (aspect ratio 1.25:1). For the CeO_2_ stamps all dimensions were stated (15 × 15 × 4 nm) and converted in a 3-parameters aspect ratio indicator (3.75:3.75:1). Ag nanowires are characterized by a high aspect ratio elongated morphology with a nominal length between 5 and 10 μm and a mean width of around 100 nm (106 ± 14 nm), having the highest aspect ratio among the NMs, ranging from 50:1 to 120:1.

**Table 2:** NMs’ physico-chemical properties in the stock preparation

Nanomaterial	Size by TEM (nm)	Morphology (aspect ratio; variable used in similarity analyses)	HDD			ζ-Potential			
			By Intensity (nm)*	By Number (nm)*	PDI*	Mean (mV)	Conductivity (mS/cm)	Dilution factor	pH
CeO_2_ NPs	4.1 ± 0.6	Spheric (1:1; 1)	5.48 ± 0.6	3.7 ± 0.3	0.250	− 34.7 ± 2.7	0.59	1:25	8.64
CeO_2_ aggregates	6.2 ± 1.3	Spheric (1:1; 1)	180.3 ± 7.0	44.8 ± 5.4	0.405	− 17.9 ± 1.0	0.03	1:40	7.95
CeO_2_ stamps	15.3 ± 2.1	Flat (3.75:3.75:1; 2.8)	102.4 ± 5.0	33.3 ± 0.6	0.129	− 25.0 ± 0.6	0.12	1:10	8.24
TiO_2_ NPs	9.4 ± 2.8	Spheric with anisotropy (1.25:1; 1.25)	69.7 ± 7.3	33.2 ± 4.0	0.362	− 48.3 ± 0.6	0.092	1:200	6.52
TiO_2_ aggregates	9.4 ± 2.8	Spheric with anisotropy (1.25:1; 1.25)	197.9 ± 6.1	53.7 ± 25.7	0.357	− 42.5 ± 0.1	0.020	1:200	6.52
TiO_2_ nanorods	L 142 ± 29	Rod (3.7:1; 3.7)	143.4 ± 2.8	87.4 ± 6.1	0.118	− 45.8 ± 1.0	0.086	1:200	10.0
	W 38.2 ± 7.9								
Ag 20 nm NPs	23.9 ± 4.4	Spheric (1:1; 1)	50.7 ± 4.0	15.0 ± 5.7	0.282	− 28.2 ± 6.7	0.04	1:125	6.71
Ag 50 nm NPs	52.9 ± 4.8	Spheric (1:1; 1)	63.4 ± 1.0	38.0 ± 2.8	0.236	− 43.3 ± 5.4	0.05	1:50	6.60
Ag nanowires	L 5–10 μm W 106 ± 14	Wire (100:1 – 50:1; 75)	1st 423 ± 177		0.226	− 10.4 ± 0.7	0.01	1:50	6.8
			2nd 5170 ± 490						

^*^Mean of 3 independent experiments ± SD

More details on NMs characterization, both for the morphology of the NMs cores and for the colloidal characterization can be found in Supplementary material 1

The NMs aggregation status and colloidal state was assessed by DLS (size by number) and ζ-potential and reported in Table 2. As expected, the CeO_2_ aggregates show larger HDD compared to their NPs counterparts, respectively 44.8 ± 5.4 nm and 3.7 ± 0.3 nm, in agreement with the nominal size reported in Table 1. The difference is less marked between TiO_2_ NPs and aggregates, where the HDD is respectively 33.2 ± 4.0 nm and 53.7 ± 25.7 nm, showing some level of aggregation in TiO_2_ NPs. UV–vis data reported in Supplementary material 1 also give information on the colloidal state, where the absence of an absorption band at wavelengths larger than 600 nm (manifesting aggregation) and the measured spectra stability over time indicate the stability of the colloids.

The ζ-Potential measurements show that all the NMs were negatively charged, with the lower values found in the Ag nanowires (− 10.4 ± 0.7 mV) and the highest values (> − 40 mV) in all the TiO_2_ NMs and the Ag 50 nm NPs (Table 2).

Size distribution and ζ-Potential analysis of the NMs in cell culture medium (DMEM/FBS) was carried out to characterize the stability of the dispersions prepared for the hazard assessment assays (Table 3). The analyses were performed shortly after the preparation of the dispersions (0 h, corresponding to the beginning of cells exposure) and after 24 h (corresponding to the end of exposure). The concentration tested was 100 µg/mL, which was the highest concentration used for the in vitro experiments.

**Table 3:** NMs physico-chemical properties in cell culture medium (DMEM/FBS)

Nanomaterial	Conc. (µg/mL)	Dilution factor	HDD*				ζ-Potential*			
			Mean size (nm) t = 0 h	PDI t = 0 h	Mean size (nm) t = 24 h	PDI t = 24 h	Mean ζ (mV) t = 0 h	Conductivity (mS/cm) t = 0 h	Mean ζ (mV) t = 24 h	Conductivity (mS/cm) t = 24 h
CeO_2_ NPs	100	1:18	75.1 ± 1.8	0.253	67.4 ± 1.3	0.247	− 9.5 ± 0.4	18.4	− 10.5 ± 0.8	18.2
CeO_2_ aggregates	100	1:26	2786 ± 627.6	0.384	2591 ± 911.1	0.217	− 9.9 ± 0.6	18.2	− 10.1 ± 1.0	17.8
CeO_2_ stamps	100	1:26	666.2 ± 46.2	0.540	462.7 ± 4.8	0.400	− 10.3 ± 0.5	18.1	− 10.6 ± 0.6	17.6
TiO_2_ NPs	100	1:25	303.8 ± 6.1	0.246	303.6 ± 5.5	0.237	− 10.7 ± 0.5	18.1	− 11.1 ± 0.9	17.6
TiO_2_ aggregates	100	1:24	233.9 ± 5.2	0.230	234.3 ± 5.6	0.203	− 10.2 ± 0.8	17.8	− 11.1 ± 1.3	17.8
TiO_2_ nanorods	100	1:26	203.1 ± 4.7	0.148	211.5 ± 4.3	0.154	− 10.9 ± 0.9	18.2	− 10.9 ± 1.2	18.1
Ag 20 nm NPs	100	1:8	61.6 ± 1.4	0.235	59.9 ± 0.9	0.230	− 7.2 ± 1.1	18.0	− 8.3 ± 1.1	18.6
Ag 50 nm NPs	100	1:18	84.4 ± 1.0	0.183	86.8 ± 1.3	0.184	− 6.6 ± 1.2	18.7	− 7.35 ± 0.1	19.3
Ag nanowires	100	1:9	258.2 ± 2.7	0.180	225.6 ± 1.1	0.170	− 3.3 ± 0.6	16.7	− 7.9 ± 0.2	17.1

^*^Mean of 3 independent experiments ± SD

The Ag 20 nm NPs, Ag 50 nm NPs, TiO_2_ aggregates and TiO_2_ nanorods displayed slightly larger HDD in DMEM/FBS than in milliQ water (stock preparations), likely due to the formation of protein corona [66]. The Ag nanowires were the only NM showing a decrease of HDD in the DMEM/FBS preparation compared to the milliQ water. TiO_2_ NPs in DMEM/FBS showed a four-times fold increase of HDD compared to dispersions in milliQ water. This could be attributed to an initial aggregation of the small-sized 8 nm particles due to their high ionic strength and to the high content of monovalent and bivalent cations in DMEM/FBS, followed by the formation of protein corona. It is also worth noting that the working concentration of TiO_2_ NPs was ten-fold higher in DMEM/FBS than in milliQ, thus increasing the chances of NP-NP interaction and aggregation in DMEM/FBS. The dispersions of CeO_2_ NPs, CeO_2_ aggregates and CeO_2_ stamps had a considerable increase of HDD in DMEM/FBS compared to dispersions in milliQ water (17-, 15- and sevenfold increase, respectively).

Once in DMEM/FBS, the mean HDDs and PDI values (PDI < 0.25) for most of the NMs did not differ over time, from the initial to the final measurements (time points 0 h and 24 h respectively), indicating the high stability of these dispersions. On the other hand, the CeO_2_ nanoforms in DMEM/FBS showed PDI values > 0.25 and the size distribution disparity from time point 0 h to 24 h was higher compared to TiO_2_ and Ag NMs. Therefore, the stability of CeO_2_ dispersions was negatively influenced by the ionic strength and near neutral pH of DMEM/FBS (~ 7.4) [4, 33]. The media’s ionic strength and protein composition were also reflected in the shift of ζ-Potential towards higher values in DMEM/FBS than in milliQ water for all NMs. The ζ-Potential and conductivity of the dispersions of TiO_2_ and CeO_2_ were uniform around -10 mV and 18 mS/cm irrespective of the size of NMs, while values for ζ-Potential of Ag NMs range from -3.3 mV to -8 mV. These values correspond to proteins in the media, indicating the formation of a protein corona coating the NMs. Taken together, ionic strength, cation and protein composition of DMEM/FBS shifted the balance of the NMs dispersions from strongly anionic to approximately neutral which might have contributed to a shift of mean-size towards larger values when compared to dispersions in milliQ water. The cell culture medium composition seemed to be particularly relevant for the stability of dispersions of CeO_2_ NMs.

### Cell-NMs interaction and uptake

TEM imaging was used to visualize cell morphology, NM uptake and localization in A549 cells (Fig. 2). The unexposed cells exhibited an oval-like shape with a well expanded cytoskeleton, and electron-dense and electron-transparent vesicles. In contrast, the cells exposed to TiO_2_ NMs were rather round in shape having a high number of filopodia at the periphery. The A549 cells internalized all three different types of TiO_2_ NMs, which mostly accumulated in large size vesicles, likely endosomes and endo-lysosomes and, in particular for TiO_2_ aggregates, within autophagic-like vacuoles. Non-membrane-enclosed clusters of TiO_2_ aggregates and nanorods could be observed in the cytoplasm. Similar to control cells, A549 exposed to CeO_2_ NMs showed a well-expanded cytoplasm. The CeO_2_ NMs were readily taken up by cells, particularly the CeO_2_ stamps. Internalized CeO_2_ NMs were detected in non-membrane- and membrane-enclosed clusters in the cytoplasm. Additionally, CeO_2_ stamps could be observed within the nucleus. Cells exposed to Ag NMs were round in shape with numerous cellular protrusions and their nuclei were irregular with several indentations. Notably, Ag nanowires inflicted severe morphological changes in the cells, which included the formation of large and vast numbers of vacuoles with features characteristic of autophagic vacuoles. In addition, all Ag NMs were internalized by A549 cells, however, their presence inside the cells was not as abundant as it was for CeO_2_ and TiO_2_ NMs. The 20 and 50 nm Ag NPs were present as single particles in the cytosol and in small endocytic vesicles, while Ag nanowires could be found inside autophagic-like vacuoles.

### Cytotoxicity

#### Alamar blue (AB)

**Fig. 3:**
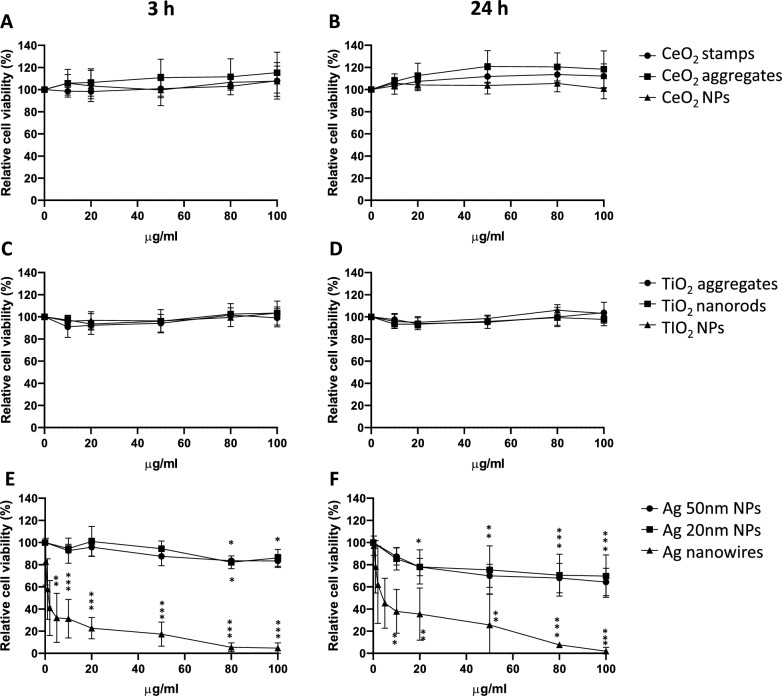
Relative cell viability (metabolic activity) measured by Alamar Blue assay in human lung epithelial A549 cells exposed to NMs for 3 (**A**, **C**, **E**) and 24 (**B**, **D**, **F**) hours. Data are presented as mean values from at least 3 independent experiments (with two technical replicates in each independent experiment) + / − SD, relative to negative control (NC). Statistically significant difference respect to NC according to one-way ANOVA with Dunnett’s post-hoc test, *p < 0.05, **p < 0.01, ***p < 0.001

An effect on cell viability/metabolic activity was observed in the cells exposed to Ag NMs, while no response was observed after TiO_2_ and CeO_2_ NMs exposure (Fig. 3). Ag 20 nm NPs and Ag 50 nm NPs showed similar response curves. A trend for concentration dependent response could be observed, however due to the shape of the curves the EC_50_ values could not be determined. The cytotoxic effect was more accentuated after 24 h of exposure compared to 3 h exposure. For the Ag 50 nm NPs the reduction of viability after 24 h exposure was statistically significant starting from the concentration 20 µg/ml, while after 3 h of exposure, statistical significance was obtained from the concentration 80 µg/ml. Ag 20 nm NPs produced a similar reduction of viability, however the effect was not statistically significant, except for the concentration 80 µg/ml after 3 h of exposure. Ag nanowires were the most toxic NMs, showing a clear concentration–response effect already after 3 h of exposure, when the reduction of viability was statistically significant starting from 1 µg/ml. After 24 h exposure the cytotoxic effect was partly reduced, with a statistically significant reduction starting from 10 µg/ml, suggesting a recovery effect. Calculated EC_50_ values for this nanoform were 1.88 ± 0.54 and 5.43 ± 1.5 µg/ml at 3 and 24 h of exposure, respectively. The results of the test’s controls are reported in supplementary material S4 Table 1, showing a statistically significant effect induced by the PC, while the dispersion medium had no effect on the cells, and no interference of the NMs with the test was observed.

#### Colony forming efficiency (CFE)

**Fig. 4:**
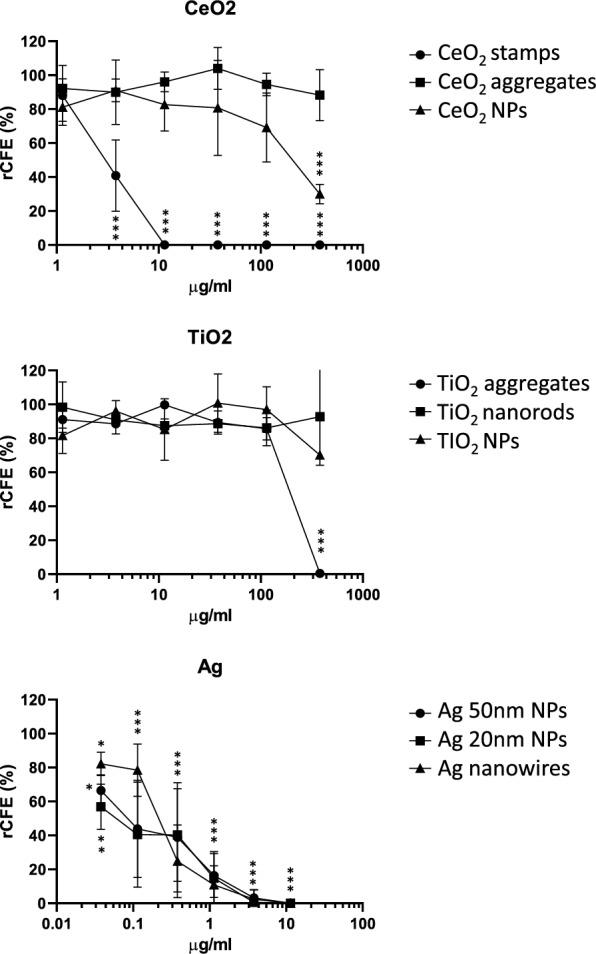
Colony forming efficiency (CFE) in human lung epithelial A549 cells after exposure to NMs. Data are presented as mean values from 3 independent experiments (with two technical replicates in each independent experiment) + / − SD, relative to negative control (NC). Statistically significant difference respect to NC according to one-way ANOVA with Dunnett’s post-hoc test, *p < 0.05, **p < 0.01, *** *p* < 0.001

An effect on cell viability was observed by the CFE assay after exposure to several of the NMs tested (Fig. 4). In agreement with the AB assay, the Ag NMs were the most cytotoxic, with a clear concentration–response effect, statistically significant already from the lowest of the concentrations tested. The CeO_2_ stamps also induced a concentration dependent effect, with a steep response curve in the concentration range 1.14–11.4 µg/ml. For CeO_2_ NPs and TiO_2_ aggregates a statistically significant effect was observed at the highest exposure concentration (380 µg/ml). No effect was observed with CeO_2_ aggregates, TiO_2_ nanorods and TiO_2_ NPs.

The EC_50_ values were calculated when possible, and they are reported in Table 4. The results of the test’s controls are reported in supplementary material S4 Table 2, showing a statistically significant effect induced by the PC, while the dispersion medium had no effect on the cells.

#### Electric cell-substrate impedance sensing (ECSIS)

**Fig. 5:**
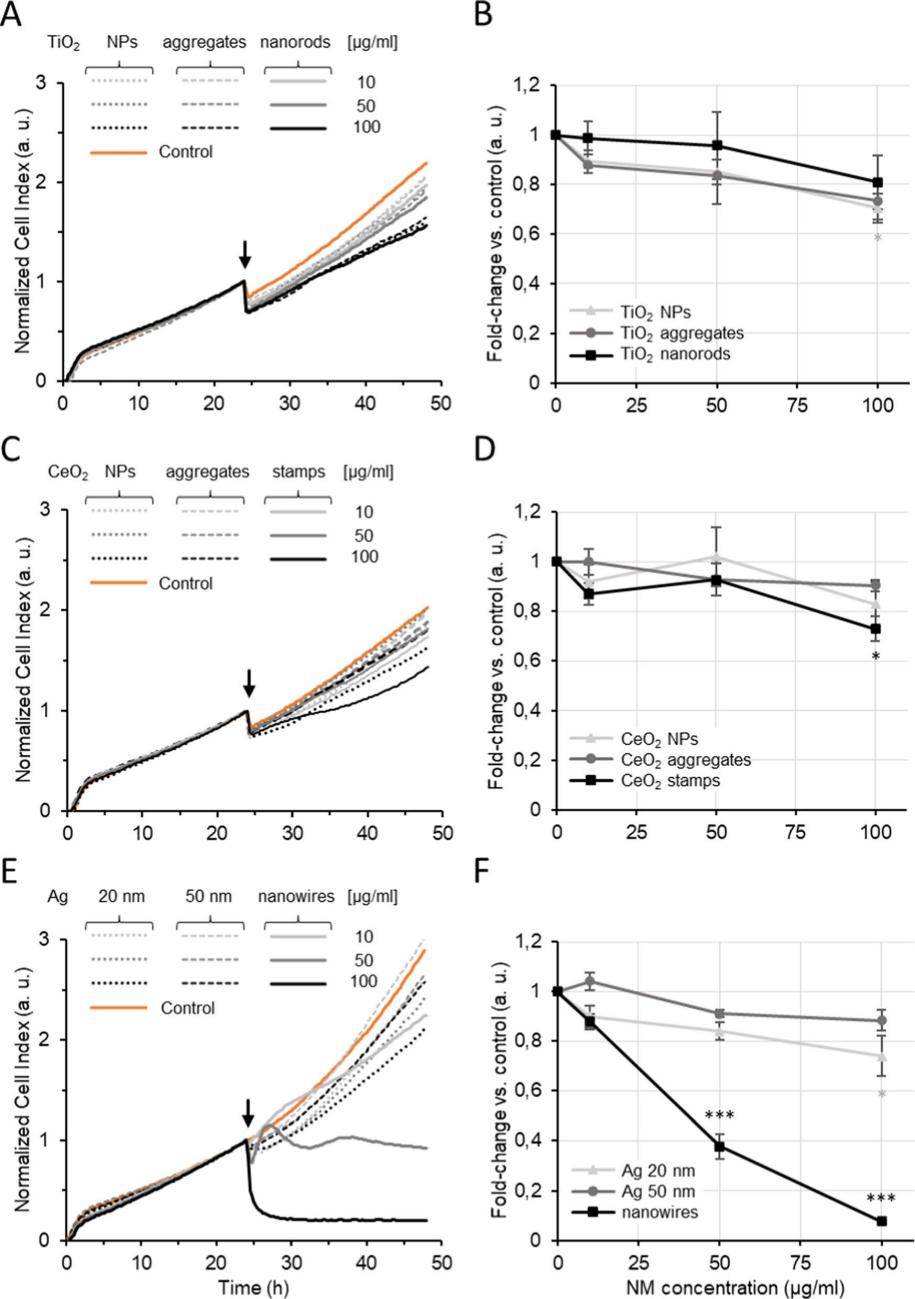
Proliferation/viability of A549 cells exposed to different NMs and analyzed by ECSIS. Cells were exposed for 24 h to TiO_2_ (**A** and **B**), CeO_2_ (**C** and **D**) or Ag (**E** and **F**) NMs at different concentrations. The data is presented as real-time cell proliferation (**A**, **C** and **E**) and fold-change vs control at the end of exposure (**B**, **D** and **F**). Arrows in **A**, **C**, and **E** indicate the beginning of exposure. Statistically significant difference respect to negative control according to one-way ANOVA with Dunnett’s post-hoc test, *p < 0.05, **p < 0.01, ***p < 0.001

ECSIS was performed to further validate the cytotoxicity data obtained by AB and CFE methods. ECSIS is a label-free technique that showed no signal interference from all the NMs tested (S4 Fig. 1). Proliferation profiles of A549 cells exposed to TiO_2_ NMs (Fig. 5a) depicted a slight fall in CI upon addition of the NMs, within 30 min. This was also visible in control (unexposed cells) conditions and was mainly caused by the temperature changes when the NM dispersions were added in the wells of the E-plates. However, the magnitude of the CI drop was higher in cells exposed to NMs than in control, suggesting an initial disruption of cell–cell and/or cell-substrate contacts caused by the presence of TiO_2_ NMs in a concentration-dependent manner. After this initial 30 min, the CI gradually increased over time at a comparable rate to that of control, except for the exposure to TiO_2_ NMs at the highest concentration (100 μg/ml); the CI rate slowed down about 10 h after addition of the NMs, which may indicate higher cell death rate in these conditions when compared to control. The fold-change vs. control was calculated at the end of the 24 h exposure duration (Fig. 5b). The TiO_2_ NMs showed a concentration- and size-dependent reduction of proliferation/viability when compared to control. The reduction was moderate and only significantly lower than control for the highest concentration tested of TiO_2_ NPs (0.70 ± 0.06 of control, *p* = 0.007). Regarding exposure to CeO_2_ NMs, the proliferation profiles of A549 cells (Fig. 5c) demonstrated a drop of CI upon addition of NMs (within 30 min), but the magnitude was comparable to control conditions. Right after, the CI steadily increased for all conditions at a similar rate, except for A549 cells exposed to 100 μg/ml of CeO_2_ NPs and particularly CeO_2_ stamps; in these conditions, the CI rate was slower compared to control. At the end of the exposure time (24 h, Fig. 5d), the relative proliferation/viability decreased marginally, with the exception of the exposure to 100 μg/ml CeO_2_ stamps (0.73 ± 0.06 of control, *p* = 0.013). Finally, we assessed the proliferation/viability of A549 cells exposed to Ag NMs (Fig. 5e). Cell growth curves showed a sharp drop and no recovery of CI upon addition of Ag nanowires at 100 μg/ml, indicating acute toxicity at this concentration. This same NM at 50 μg/ml caused a moderate drop of CI, followed by recovery and ultimately steady drop, suggesting that cells might have been able to cope with the initial stress only for a few hours. Ultimately, Ag nanowires were highly cytotoxic at concentrations ≥ 50 μg/ml (p < 0.001) with EC_50_ of 37.03 ± 6.47 μg/ml. The Ag 50 nm NPs did not seem to affect cell growth (Fig. 5e, f), while Ag 20 nm NPs showed a concentration-dependent decrease in CI rate and relative viability/proliferation at the end of exposure (0.74 ± 0.08 of control at 100 μg/ml, *p* = 0.02).

#### Comparison of cytotoxicity results and NMs categorization

Whenever possible, the EC_50_ values were calculated for the different cytotoxicity methods and the NMs were categorized as non-toxic, slightly toxic, or toxic based on the scoring system described in El Yamani et al. [21]. Table 4 reports a comparison of the results for the three cytotoxicity methods here used. Ag nanowires were the most cytotoxic NM in all the test methods, and the only material for which it was possible to calculate EC_50_ with the AB and ECSIS assays. With the CFE assay more NMs showed an effect, making this the most sensitive method among the ones used.

**Table 4:** NMs’ EC_50_ (µg/ml, average ± SEM) and categorization for cytotoxicity

Nanoform	ECSIS		AB				CFE	
	24 h		3 h		24 h		9–12 days	
	EC_50_	Categorization	EC_50_	Categorization	EC_50_	Categorization	EC_50_	Categorization
TiO_2_ NPs	NA	Non-toxic	NA	Non-toxic	NA	Non-toxic	NA	Non-toxic
TiO_2_ aggregates	NA	Non-toxic	NA	Non-toxic	NA	Non-toxic	159.32 ± 66.91	Slightly toxic
TiO_2_ nanorods	NA	Non-toxic	NA	Non-toxic	NA	Non-toxic	NA	Non-toxic
CeO_2_ NPs	NA	Non-toxic	NA	Non-toxic	NA	Non-toxic	199.76 ± 94.26	Slightly toxic
CeO_2_ aggregates	NA	Non-toxic	NA	Non-toxic	NA	Non-toxic	NA	Non-toxic
CeO_2_ stamps	NA	Non-toxic	NA	Non-toxic	NA	Non-toxic	3.11 ± 0.22	Toxic
Ag 20 nm NPs	NA	Non-toxic	NA	Non-toxic	NA	Non-toxic	0.47 ± 0.05	Toxic
Ag 50 nm NPs	NA	Non-toxic	NA	Non-toxic	NA	Non-toxic	0.43 ± 0.08	Toxic
Ag nanowires	37.03 ± 6.47	Toxic	1.88 ± 0.54	Toxic	5.43 ± 1.5	Toxic	0.07 ± 0.01	Toxic

#### Genotoxicity

**Fig. 6:**
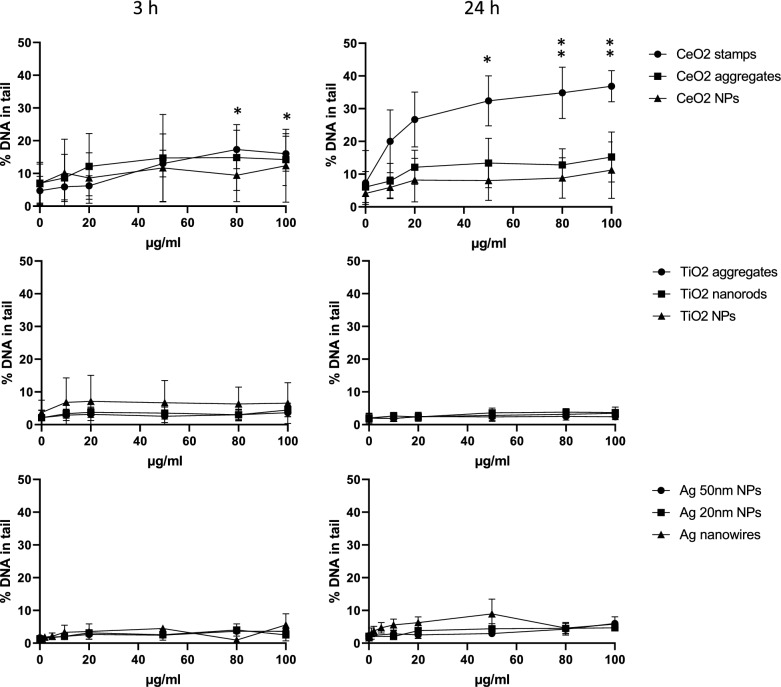
DNA damage (SBs) in A549 cells measured by the CA as percentage of DNA in tail after 3 and 24 h exposure to NMs. Data are presented as mean values from at least 3 independent experiments (with two technical replicates in each independent experiment) + / − SD. Statistically significant difference of the exposed samples compared to the negative control calculated by one-way ANOVA (Dunnett`s multiple comparisons test): *p < 0.05, **p < 0.01

**Fig. 7:**
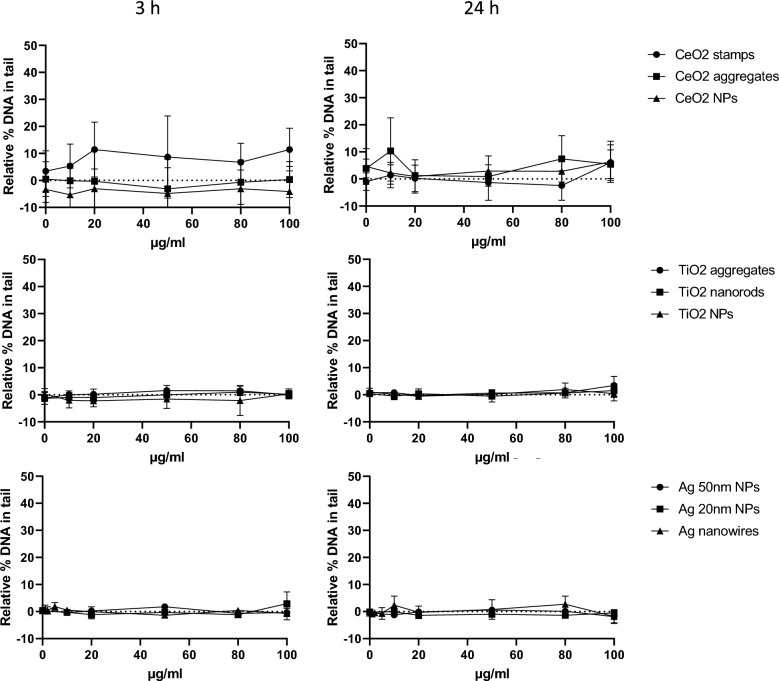
Oxidative DNA damage (net Fpg) in A549 cells measured by the CA as relative percentage of DNA in tail after 3 and 24 h exposure to NMs. Negative values represent the assay’s variability. Data are presented as mean values from at least 3 independent experiments (with two technical replicates in each independent experiment) + / − SD. Statistically significant difference of the exposed samples compared to the negative control calculated by one-way ANOVA (Dunnett`s multiple comparisons test): *p < 0.05, **p < 0.01

The NMs’ genotoxicity was assessed by the comet assay (CA). The CeO_2_ stamps were the only NM showing a clear genotoxic effect, with an increase of SBs already present after 3 h of exposure (statistically significant different from control from the concentration 80 µg/ml), and further enhanced after 24 h of exposure (statistically significant from 50 µg/ml, Fig. 6). Although a slight increase in net Fpg could be observed after 3 h of exposure of CeO_2_ stamps, this effect was not statistically significant (Fig. 7), and no increase in net Fpg was observed after 24 h. No statistically significant increase in SBs or net Fpg was observed with the other NMs at the exposure concentration tested. However, the concentration–response relationship was assessed, and a statistically significant linear trend was found for several NMs as reported in Table 5. Based on these results, the NMs were categorized for their genotoxic effect according to the scoring system described in El Yamani et al. [21] The only NM with a positive genotoxic effect was CeO_2_ stamps, which reported more than two tested concentrations significantly different from the control and a significant linear trend (Fig. 6 and Table 5). Equivocal results were reported for TiO_2_ aggregates (based on net Fpg at 24 h only), TiO_2_ nanorods (based on both SBs and net Fpg), CeO_2_ aggregates and all the Ag nanoforms (SBs only), due to the fact that a significant linear trend was found for these NMs, but none of the tested concentrations gave a significant increased DNA damage over the level of control.

**Table 5:** Statistical significance of the DNA damage effect and categorization for genotoxicity

Nanoform	SBs				net Fpg			
	3 h		24 h		3 h		24 h	
	Significance	Categorization	Significance	Categorization	Significance	Categorization	Significance	Categorization
TiO_2_ NPs	No	Negative	No	Negative	No	Negative	No	Negative
TiO_2_ aggregates	No	Negative	No	Negative	No	Negative	*p*: 0.04#	Equivocal
TiO_2_ nanorods	No	Negative	*p*: 0.006#	Equivocal	*p*: 0.004#	Equivocal	No	Negative
CeO_2_ NPs	No	Negative	No	Negative	No	Negative	No	Negative
CeO_2_ aggregates	No	Negative	*p*: 0.048#	Equivocal	No	Negative	No	Negative
CeO_2_ stamps	2*; *p*: 0.0001#	Positive	3*; *p*: 0.001#	Positive	No	Negative	No	Negative
Ag 20 nm NPs	No	Negative	*p*: 0.009#	Equivocal	No	Negative	No	Negative
Ag 50 nm NPs	*p*: 0.002#	Equivocal	*p*: 0.001#	Equivocal	No	Negative	No	Negative
Ag nanowires	*p*: 0.024#	Equivocal	*p*: 0.020#	Equivocal	No	Negative	No	Negative

Significance: *Statistically significant increase with respect to control for the number of concentrations indicated, calculated by one-way ANOVA (Dunnett’s multiple comparisons test); #Statistically significant linear trend (*p* < 0.05) calculated by linear regression analysis (slope significantly non-zero)

#### Similarity analyses

**Fig. 8:**
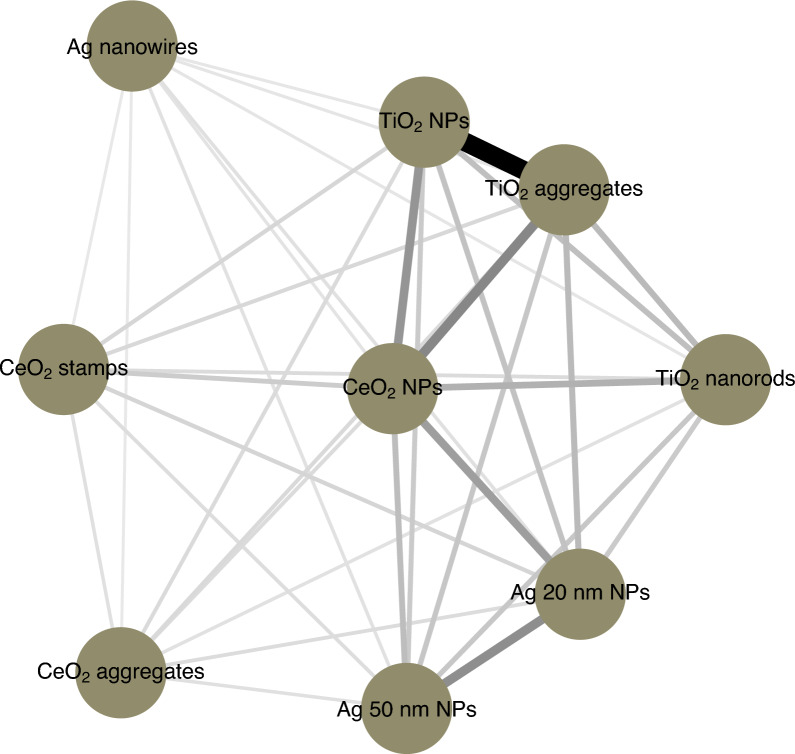
Connectivity graph. The color intensity and width of the edges indicate the similarity level of NMs in terms of Euclidean distance

To identify the similarity among NMs and gain an understanding of the relationships and connections between the studied particles, the similarity analysis was conducted based on the NMs physico-chemical properties using the network approach. In essence, network modeling techniques seek to visualize the connectivity relationship between objects (here NMs) as a network in which each particle is a node and each distance/similarity measure is an edge. The color intensity and width of the edges indicate the similarity level of NMs in terms of Euclidean distance. Very thin and almost transparent edges mean low similarity, whereas very wide and dark edges mean particles are more alike. To study particle (dis)similarity, a 11-dimensional space of NMs’ physico-chemical properties, i.e., the nominal sizes, size distribution, polydispersity index, ζ-potential, aspect ratio etc., were used. As evident from Fig. 8, TiO_2_ NPs and TiO_2_ aggregates appear to be the most similar to each other (indicated by the shortest distance and darkest colors in the connectivity graph). Conversely, Ag nanowires are the most distant from all other studied NMs.

**Fig. 9:**
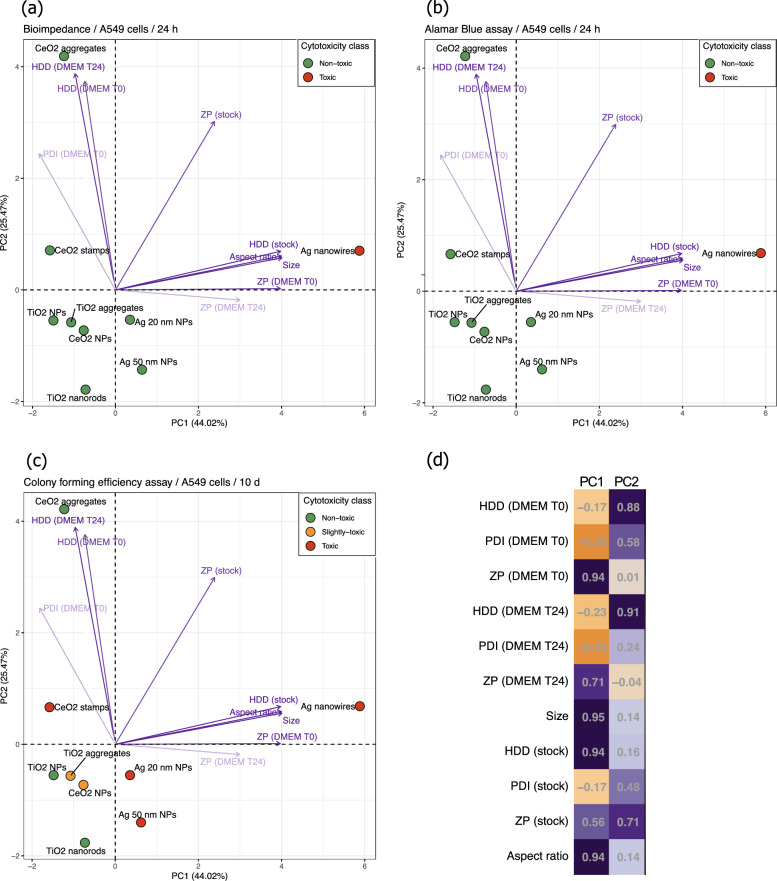
The results of the PCA analysis: **a** Electric cell-substrate impedance sensing (ECSIS) in human lung epithelial A549 cells exposed to NMs for 24 h; **b** Alamar blue (AB) assay in human lung epithelial A549 cells exposed to NMs for 24 h; **c** Colony forming efficiency (CFE) in human lung epithelial A549 cells exposed to NMs for 9–12 days; **d** The plot of the normalized factor loadings. ZP: ζ-Potential; Size: crystalline size of the NMs measured by TEM; HDD: hydrodynamic diameter in stock (milliQ water) or DMEM (cell culture medium), at time point 0 h (T0) or 24 h (T24); PDI: polydispersity index

To simultaneously identify the associations between the NMs’ cytotoxicity and their physico-chemical properties covering the morphology of the inorganic NMs core, their intrinsic properties and colloidal state, a principal component analysis (PCA) was applied. The first two principal components explain jointly 69.49% (44.02% + 25.47%) of the total variance in the data and clearly show the NMs’ clustering according to their physico-chemical properties and cytotoxicity differences. Although some differences depending on the exposure time and/or assay used may be observed, the overall conclusion is common. Projection of the data onto the subspace defined by the first principal component (PC1) and the second principal component (PC2), shown as a biplot in Fig. 9, provides insight into the relationship between NMs cytotoxicity and their physicochemical properties. The non-toxic particles are located on the left side of the biplot; while moving along the x-axes (i.e., PC1), the particles change from non-toxic through slightly toxic in the case of CFE assay, to toxic. To explore the mechanistic links between NM cytotoxicity and these properties, the normalized factor loadings were analyzed (Fig. 9d). These loadings represent correlations between the original variables and the principal components, quantifying how much each variable contributes to a given principal component. According to Malinowski’s rule, only loadings with absolute values of 0.7 or greater are statistically significant. As shown in Fig. 9d, the variables that most strongly influence PC1 are size, followed by aspect ratio, surface charge (ζ-potential) measured right after preparation (T0) and 24 h after preparation (T24), and hydrodynamic diameter (HDD) in the stock solution. All these variables are positively correlated with PC1, indicating that NMs with low PC1 values (x-axis) have low values for these variables, while those with high PC1 values have high values. PC2, on the other hand, is primarily associated with HDD in cell culture medium measured at T0 and T24, as well as ζ-potential in stock solution (Fig. 9d).

The results of PCA for electric cell-substrate impedance sensing (ECSIS) in human lung epithelial A549 cells exposed to NMs for 24 h (Fig. 9a) and the Alamar Blue (AB) assay in A549 cells exposed to NMs for 24 h (Fig. 9b) are relatively straightforward and intuitive. However, for the more sensitive colony-forming efficiency (CFE) assay, which categorizes NM toxicity as “non-toxic”, “slightly toxic”, or “toxic”, the PCA analysis could not clearly distinguish these categories (Fig. 9c). More specifically, although all silver nanoforms (Ag nanowires, Ag 20 nm NPs, and Ag 50 nm NPs) are classified as “toxic” in the CFE assay, the PCA does not clearly indicate similarities between these nanoforms. This is because the distance between the points (NMs) on the PCA plot represents their degree of similarity: points that are close to each other have similar profiles (i.e., similar physico-chemical properties and are therefore expected to have similar biological activities, such as cytotoxicity), while points that are farther apart have dissimilar profiles.

**Fig. 10:**
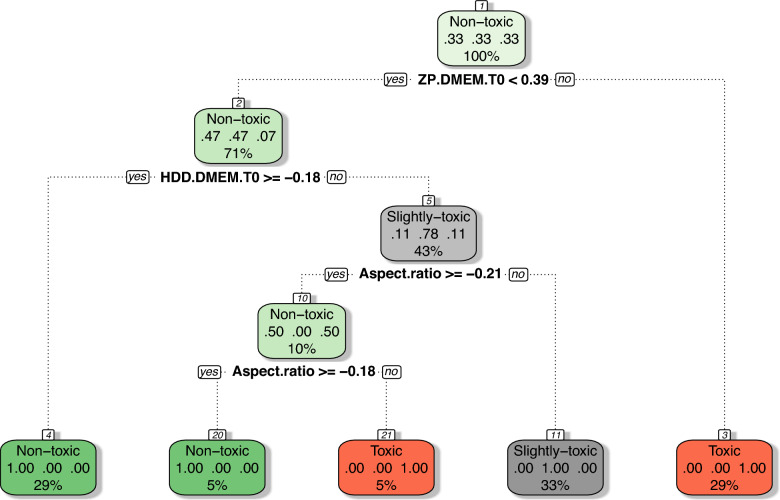
Classification tree (CT) model for cytotoxic responses in the CFE assay for human lung epithelial A549 cells. The CT model performs recursive binary splitting, meaning each node divides the data into two subsets based on a single feature. At each internal node, a decision rule is applied: if the condition is met (the answer is “*yes*”), the data is directed to the left child node; otherwise (the answer is “*no*”), the data is directed to the right child node. For example, at the root node, the decision rule ‘ZP DMEM T0 < 0.39’ means that the left branch corresponds to data (i.e., NMs) that satisfies this condition, while the right branch represents data (i.e., NMs) where ‘ZP DMEM T0’ is greater than or equal to 0.39. Toxic nanomaterials are marked in red, slightly toxic in grey, and non-toxic in green. Each node displays: (1) the predicted toxicity class (toxic, slightly toxic, or non-toxic), (2) the predicted probability for each class, and (3) the percentage of observations within the node. It is important to note that the CT model utilizes auto-scaled feature values. Therefore, a ‘ZP DMEM T0’ value of 0.39 corresponds to − 8.329 (mV), a ‘HDD DMEM T0’ value of -0.18 corresponds to 247.32 (nm), and ‘Aspect ratio’ values of − 0.21 and − 0.18 correspond to 1.652 and 2.789, respectively. It is also worth noting that a single feature may appear multiple times within a given CT model

Instead, Ag 20 nm NPs and Ag 50 nm NPs appear to be closer to certain slightly toxic or non-toxic materials, such as CeO_2_ NPs and TiO_2_ nanorods, respectively. To address this limitation and to evaluate the effectiveness of in silico methods in accurately classifying NMs based on their physico-chemical properties, we next applied structure–activity relationship (SAR) modeling using one of the most popular and intuitive approaches: classification tree (CT). CT facilitates the construction of predictive models by identifying the most relevant predictors for the target variable, leading to a more efficient classification into different toxicity classes. The CT algorithm uses a top-down approach, recursively partitioning the data into homogeneous subsets (known as “child nodes”). The process starts with the “root node” (the entire training data set) and selects the variable most strongly associated with the response variable (in this case, cytotoxicity class) to initiate the partitioning. The algorithm continues to divide the data into increasingly pure subsets by selecting variables at each node based on the lowest impurity measure (Gini index) among all properties. This process continues until the “leaf nodes” are reached, which provide the final classification results for making predictions on new (unseen) data [28, 52]. Major limitations of in silico models, including CTs, are the limited availability of data and the challenge of achieving a balanced dataset, which can lead to bias toward the majority class and/or reduce the reliability of classifications. To mitigate the imbalance problem, techniques such as undersampling (removing samples from the majority class) or oversampling (increasing samples in the minority class) can be used. Given the limited number of representatives in each cytotoxicity class—“toxic” with 4 NMs, “slightly toxic” with 2, and “non-toxic” with 3—we opted for the oversampling technique. Specifically, the Synthetic Minority Over-sampling Technique (SMOTE) was applied, which synthetically generates new samples for minority classes in the feature space (independent variables), thereby improving class balance and enhancing model robustness [14]. Using the SMOTE technique, the sample size for each of the three cytotoxicity classes was increased to 10, resulting in a data set of 30 samples. In line with standard in silico modeling practices, each model is trained on a designated training set and then validated on an external test set to assess its predictive performance. Therefore, before developing the CT model, the dataset of 30 NMs was divided into a training set of 21 samples and a test set of nine samples. This division was structured so that all NMs originally used in the study were included in the training set, supplemented with synthetically generated data to balance each cytotoxicity class to seven samples. The test set contained three samples from each of the three cytotoxicity classes. Following this data preparation, a classification tree model was developed to predict cytotoxic responses in the CFE assay for human lung epithelial A549 cells. The resulting CT model (Fig. 10) relies on three descriptors to classify NMs as toxic, slightly toxic, or non-toxic: ‘ZP DMEM T0’ (the surface charge (ζ-Potential)), ‘HDD DMEM T0’ (HDD in cell culture medium measured right after preparation), and aspect ratio. Remarkably, the CT model correctly classified all NMs in both the training and test sets (Table 6), yielding the following performance metrics: accuracy of 100%, sensitivity of 100%, specificity of 100%, precision of 100%, and an error rate of 0%. These performance metrics are noteworthy, as models with accuracy levels of 70% or higher are generally considered acceptable in practice. In addition, to assess whether the model’s performance was affected by the specific split and composition of the training and test sets, the modeling process was repeated with different dataset splits. Notably, the model consistently achieved 100% accuracy in both training and validation, regardless of the dataset split, underscoring the robustness of the model.

**Table 6:** Details of the classification tree (CT) model developed to predict cytotoxic responses in the colony forming efficiency (CFE) assay for human lung epithelial A549 cells

Nanoform	ZP DMEM T0	HDD DMEM T0	Aspect ratio	Set	Cytotoxicity (CFE assay)		Correctness
					Observed	Predicted	
Ag nanowires	− 3.29	258.20	175.00	T	Toxic	Toxic	TRUE
Ag 50 nm NPs	− 6.64	84.41	1.00	T	Toxic	Toxic	TRUE
Ag 20 nm NPs	− 7.19	61.55	1.00	T	Toxic	Toxic	TRUE
CeO_2_ stamps	− 10.30	66.20	2.00	T	Toxic	Toxic	TRUE
SMOTE sample data-1	− 6.93	72.34	1.00	T	Toxic	Toxic	TRUE
SMOTE sample data-2	− 7.13	64.01	1.00	T	Toxic	Toxic	TRUE
SMOTE sample data-3	− 6.94	71.91	1.00	T	Toxic	Toxic	TRUE
CeO_2_ NPs	− 9.46	75.09	1.00	T	Slightly-toxic	Slightly-toxic	TRUE
TiO_2_ aggregates	− 10.20	233.90	1.25	T	Slightly-toxic	Slightly-toxic	TRUE
SMOTE sample data-4	− 10.16	224.45	1.24	T	Slightly-toxic	Slightly-toxic	TRUE
SMOTE sample data-5	− 9.49	82.32	1.01	T	Slightly-toxic	Slightly-toxic	TRUE
SMOTE sample data-6	− 9.81	150.03	1.12	T	Slightly-toxic	Slightly-toxic	TRUE
SMOTE sample data-7	− 9.79	146.33	1.11	T	Slightly-toxic	Slightly-toxic	TRUE
SMOTE sample data-8	− 9.76	140.04	1.10	T	Slightly-toxic	Slightly-toxic	TRUE
TiO_2_ NPs	− 10.70	303.80	1.25	T	Non-toxic	Non-toxic	TRUE
TiO_2_ nanorods	− 10.90	203.10	3.70	T	Non-toxic	Non-toxic	TRUE
CeO_2_ aggregates	− 9.93	2786.00	1.00	T	Non-toxic	Non-toxic	TRUE
SMOTE sample data-9	− 10.71	299.21	1.36	T	Non-toxic	Non-toxic	TRUE
SMOTE sample data-10	− 10.79	258.63	2.35	T	Non-toxic	Non-toxic	TRUE
SMOTE sample data-11	− 10.67	410.95	1.24	T	Non-toxic	Non-toxic	TRUE
SMOTE sample data-12	− 10.28	1660.73	1.11	T	Non-toxic	Non-toxic	TRUE
SMOTE sample data-13	− 6.66	83.42	1.00	V	Toxic	Toxic	TRUE
SMOTE sample data-14	− 3.44	250.28	167.07	V	Toxic	Toxic	TRUE
SMOTE sample data-15	− 8.28	76.24	1.45	V	Toxic	Toxic	TRUE
SMOTE sample data-16	− 10.11	215.32	1.22	V	Slightly-toxic	Slightly-toxic	TRUE
SMOTE sample data-17	− 9.54	92.17	1.03	V	Slightly-toxic	Slightly-toxic	TRUE
SMOTE sample data-18	− 9.86	161.39	1.14	V	Slightly-toxic	Slightly-toxic	TRUE
SMOTE sample data-19	− 10.89	209.09	3.55	V	Non-toxic	Non-toxic	TRUE
SMOTE sample data-20	− 10.79	256.28	2.41	V	Non-toxic	Non-toxic	TRUE
SMOTE sample data-21	− 10.72	292.97	1.51	V	Non-toxic	Non-toxic	TRUE

T and V indicate training and test sets, respectively

**Fig. 11:**
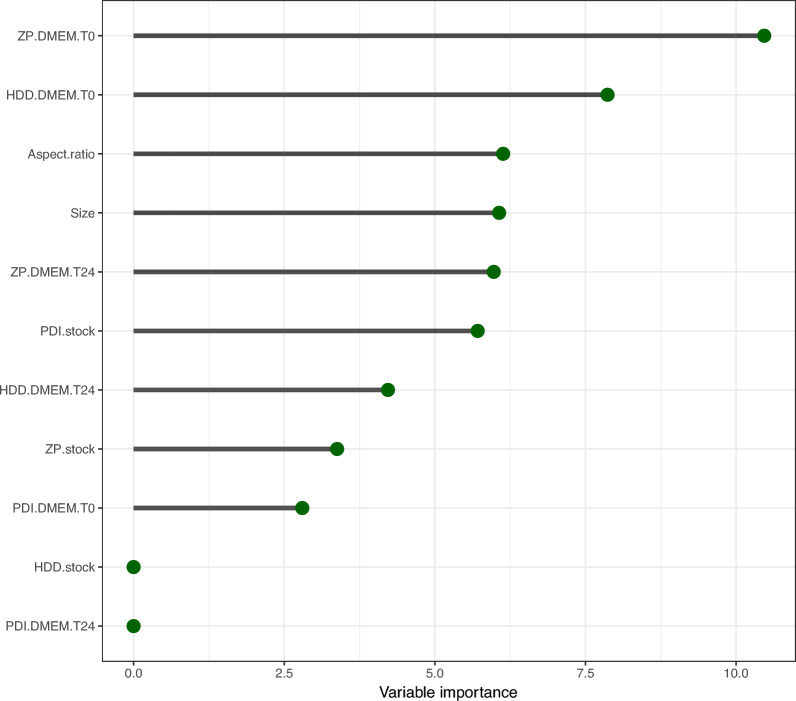
Variable importance plot from a classification tree (CT) model for cytotoxic responses in the CFE assay for human lung epithelial A549 cells

To identify the most influential features in the CT model, a variable importance plot was analyzed. This plot provides a visual ranking of the importance of each feature in the classification model, with the most important features listed at the top and the least important at the bottom. As depicted in Fig. 11, the most important structural parameter for classifying NMs was ‘ZP DMEM T0’, which distinguished silver nanomaterials with the highest surface charge (ζ-potential) measured right after preparation (T0) (i.e. ‘ZP DMEM T0’ >  − 8.329 (mV)) from other NMs. The second most influential feature was ‘HDD DMEM T0’. Nanomaterials with a ζ-potential <  − 8.329 mV and a sufficiently large hydrodynamic diameter in cell culture medium (DMEM) at time 0 h (T0) (> 247.32 nm), such as TiO_2_ nanoparticles and CeO_2_ aggregates, were classified as non-toxic at this stage. The third most important feature was the aspect ratio. In general, nanomaterials with a high surface-to-volume ratio tend to exhibit increased surface energy and/or activity, which often play a critical role in cellular uptake. Interestingly, the mean size was found to be of comparable importance to the aspect ratio in the CT model, further highlighting the importance of structural features in determining the cytotoxicity of NMs.

The results of the CT model (Figs. 10 and 11) supported previous findings from the interpretation of normalized factor loadings in the PCA analysis (Fig. 9d), highlighting the importance of variables such as surface charge measured in DMEM, the HDD of NMs colloids, the mean size (crystalline size measured by TEM), and the aspect ratio in distinguishing NMs based on their cytotoxicity. This observation was further confirmed by two-way hierarchical cluster analysis (2D-HCA). This approach combines a dendrogram with a heatmap to visualize the clustering of NMs by their properties and the clustering of physico-chemical properties. The 2D-HCA, as a pattern recognition algorithm, relies on distance metrics that quantify the similarity between variables/observations. Herein Euclidean distance as the similarity measure and Ward linkage as the agglomeration method were used to assess the similarity patterns in the data.

**Fig. 12:**
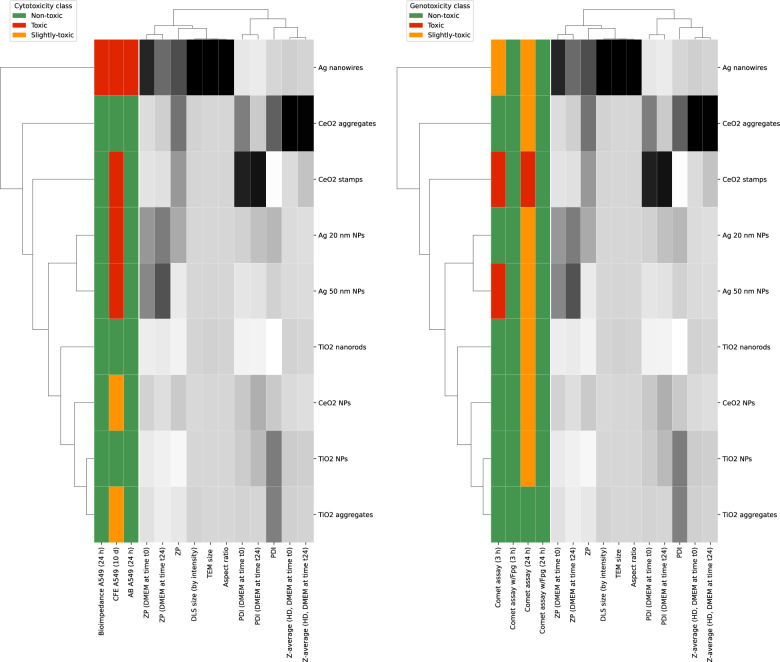
Two-way hierarchical cluster analysis (2D-HCA) for cytotoxicity and genotoxicity. Branches in the dendrograms correspond to the relative degree of similarity among the variables/observations: the shorter the branch, the higher the degree of similarity; the greater the branch, the greater dissimilarity. ZP: ζ-Potential; Size: crystalline size of the NMs measured by TEM; HDD: hydrodynamic diameter in stock (milliQ water) or DMEM (cell culture medium), at time point 0 h (T0) or 24 h (T24); PDI: polydispersity index. *AB* alamar blue assay; *CFE* colony forming efficiency assay; *ECSIS* electric cell-substrate impedance sensing assay

A two-way hierarchical cluster analysis (Fig. 12) confirmed that Ag nanowires with the greatest size measured by TEM and HDD size measured by DLS, aspect ratio and surface charge are farthest in the distance from other NMs, thereby, seem to differ from other studied particles. The second most distant particle is CeO2 aggregates. This can be explained by the fact that this particle is characterized by the highest values of the surface charge (ζ-Potential), and HDD in cell culture medium measured right after preparation (T0) and 24 h after preparation (T24). Interestingly, higher cytotoxic responses were generally observed for the CFE assay in human lung epithelial A549 cells than for the other two assays after exposure to NMs. The CFE assay was found to be more sensitive to NMs than ECSIS or AB assays. A possible explanation for differing responses to NM exposure is the exposure time (9–12 days for CFE vs. 24 h for AB and ECSIS). Besides, while in the AB and ECSIS assays the cells are exposed while they lay in a monolayer, single cells are exposed to the NMs in the CFE assay. This might influence the cells behavior and sensitivity to exposure. Another possible explanation is that the assays used in this study investigate a wide range of mechanisms that provide specific response(s), as further addressed in the discussion.

## Discussion

In this work we combine the use of in vitro assays, a thorough physico-chemical characterization, and in silico analyses to address the hazard assessment of NMs in view of NGRA, taking into consideration the specific challenges posed by NMs.

Different nanoforms representing widely used metal and metal oxide NMs were selected for the study. The TiO_2_ NMs are the most highly produced worldwide and used in many industrial and cosmetic products [64], while the CeO_2_ NMs, in addition to being a mature in the petrochemical and polymers industry, are getting increasing attention in healthcare due to their potential uses in nanomedicine [12, 68]. The Ag NMs are the most widely produced example of a metal (oxidation number 0) NM, with applications related to their antimicrobial properties in medical devices, wound dressings, drug delivery, and industrial applications like coatings, textiles, and electronics. For each of these substances, three different morphologies and/or colloidal states were used, for a total of nine different nanoforms. The role of the intrinsic physical properties of the materials, their aggregation status, besides their chemical composition, in eliciting toxicological responses was thus investigated.

As the dispersion and sedimentation behavior of NMs is known to affect the dosimetry and thus the biological responses elicited by NMs [43, 46], the evolution of the NMs in the working media, including their aggregation and stability, were thoroughly examined. The stability and size distribution of the nanoforms was assessed through TEM, UV–vis and DLS analyses at both the beginning and end of the experiments. The stability of the NMs in the culture medium used throughout the duration of the experiments was thus confirmed. Additionally, the internalization and interactions of cells with the particles were evaluated via TEM analyses of exposed cells. This clearly showed that the lack of toxicity of some of the NMs was not due to lack of cell-particles interaction or particles’ deposition. These comprehensive assessments support the findings here reported and provide a robust framework for understanding the correlation between the NMs’ concentrations used and toxicity observed.

In silico analyses are powerful tools in toxicology, but still knowledge is needed when it comes to their applicability to NMs. Here we applied exploratory data analysis and SAR modelling to investigate the associations between the NMs toxicity and their physico-chemical properties. Mimicking physiologically relevant conditions, including proper exposure routes, is essential for advancing nanomaterial hazard assessment. Previous studies have emphasized the use of simulated fluids for NMs dispersion, such as simulated lung fluids for inhalation or digestion fluids for ingestion studies, [2, 31], to better reflect real-life exposure. It has to be noted that, standardized or accepted simulated lung fluids are still missing [31]. Similarly, advanced in vitro models, such as the air–liquid interface (ALI) culture models for inhalation, are considered more relevant compared to the traditional models, e.g. submerged lung cells, as they more closely mimic in vivo conditions, allow for better cellular differentiation and functions, which enhances the physiological relevance of experimental results [10]. However, advanced models have limitations concerning low throughput and less reproducible results, making them less suitable for screening phases. Interestingly, the cytotoxicity of TiO_2_ NMs in lung epithelial cells was reported to be similar in ALI or submerged cell cultures [44], suggesting that simple submerged models could be predictive enough for early screening phases of hazard assessment, and in a SSbD perspective. Here we apply the commonly used and well characterized A549 alveolar epithelial cell line in a high throughput experimental setting. The particles used were designed with controlled stability and aggregation, requiring minimal handling, thus no elaborated dispersion procedures were used, not to affect the NMs’ properties. This approach allowed us to demonstrate the applicability of in silico models in distinguishing particles based on these engineered characteristics, serving as a proof of concept for their predictive power.

It is known that nanoforms of the same element can present different toxicological behaviors. In this respect, the most notable result here observed is the genotoxic response induced by CeO_2_ stamps. Genotoxicity was assessed by the in vitro comet assay (+ / − Fpg for detection of oxidized bases), assessing the induction of DNA damage in A549 cells exposed to NMs for 3 and 24 h. At both the time points investigated, CeO_2_ stamps induced an increase in DNA strand breaks (SB), while no increase in oxidized bases was observed. This suggests that genotoxicity is induced by CeO_2_ stamps by mechanisms that do not involve oxidative stress. A genotoxic effect was not observed with the other CeO_2_ NMs, nor with any other of the materials tested. Here it was crucial to assess the cell/NMs interaction, to exclude false negatives due to NMs not being in contact with the cells [20]. TEM analyses of cells exposed to 50 μg/ml of the NMs for 24 h confirmed that all the NMs were taken up by the cells, in the cytosol and/or in membrane-enclosed organelles. Most interestingly, CeO_2_ stamps could be observed within the cell nuclei suggesting that a mechanism of direct damage to the DNA might be at least partly involved in the genotoxic effect of this nanoform.

Since of all the tested nanoforms only CeO_2_ stamps were genotoxic, there were limitations on the applicability of similarity analyses to this endpoint, and the PCA could not be performed. The 2D-HCA was run, but no strong association of genotoxicity with any of the physico-chemical parameters here investigated was detected. A weak association was found with PDI in cell culture medium, which is an indication of the NM size distribution with no obvious implication for toxicity. The CeO_2_ stamps used in this study are rather flat particles, showing some degree of anisotropy as described by their aspect ratio (3.75:3.75:1). In support to our findings, it has been previously reported that higher values of particles length and aspect ratio (> 200 nm and > 22 respectively) are needed to induce toxicity of CeO_2_ NMs [32] than the ones here observed for CeO_2_ stamps (15.3 ± 2.1 nm and 3.75 respectively). Thus, none of the physico-chemical parameters here considered seems to be able to explain or predict the CeO_2_ stamps’ genotoxic effect.

It is important to highlight that CeO_2_ stamps were the only nanoform produced in the presence of HMT, which might have toxic properties. Indeed, HMT-CeO_2_ NMs have been previously reported to present higher toxicity compared to other CeO_2_ nanoforms, as well as enhanced cellular uptake and specific catalytic properties [18]. It is well known that detergent-like molecules, or cationic amphipathic molecules, such as HMT perturb cell membranes [3], which might explain the higher cellular uptake previously reported. Thus, the presence of toxic moieties of HMT in the CeO_2_ stamps formulation might be responsible for the effects here observed, by enhancing cellular uptake, facilitating the particles to reach the nuclei and damage the DNA.

Interestingly, HMT was reported to alter the valence state of Ce [6], and in a previous study the percentage of surface content of Ce3 + sites was found to be the main driver of CeO_2_ nanoparticles toxicity [51]. This is not the case here, as the results in Supplementary material 2 show a Ce3 + content very similar in CeO_2_ stamps and aggregates, and lower compared to CeO_2_ NPs. However, the valence state of NMs might be a relevant parameter to be considered in further investigations.

The use of different methods to assess the same endpoint is a recommendation for RA of NMs [20], and it can be a way to support the implementation of NAMs in RA. In this perspective, here we assessed cytotoxicity by three assays that rely on diverse cellular functions, i.e. the AB assay based on the cellular metabolic competence and relative cell growth, the CFE based on the ability of the cells to survive/proliferate and form colonies, and the ECSIS based on relative cell growth (cell surface coverage) and cell membrane integrity. The latter two have the advantage of being label-free methods, and thus less prone to interferences caused by NMs. It has to be noted that there was a longer-term, i.e. 9–12 days, exposure of cells to NMs in the CFE assay, while in the AB and ECSIS assays, this exposure was short and lasted only 24 h. The AB and ECSIS results reported here were very consistent, with minor differences likely related to the biological process involved in the tests, which can be explained by the same duration of exposure. All the Ag nanoforms induced an effect on the viability of exposed cells, although Ag nanowires were by far the most cytotoxic according to all the methods performed. Interestingly, the TEM investigation of cells-NMs interaction showed that the cell morphology was greatly affected in the samples exposed to Ag nanowires, with an extensive formation of vacuoles, a sign connected to the process of cell death/necrosis [57]. The ECSIS assay seems to be slightly more sensitive compared to the AB assay when it comes to the TiO_2_ NPs and CeO_2_ stamps, as it detected a statistically significant decrease in cell viability/proliferation for these NMs at the highest exposure concentration, which was not picked up by the AB assay. Oppositely, the AB assay seems more responsive in the case of Ag nanoforms in general, as shown by the EC_50_ values of the Ag nanowires and the response curves of the Ag 20 nm NP and Ag 50 nm NPs. Nonetheless, according to the categorization criteria described in El Yamani et al. [21] and here applied, both the ECSIS and the AB assays results lead to the classification of TiO_2_ NPs and CeO_2_ stamps as non-cytotoxic. Being label-free, the ECSIS might be a good alternative in case of NMs that interfere with colorimetric assays. One additional possible consideration is that the ECSIS assay can be more informative compared to other assays, such as AB and CFE in this case, as it can monitor the cells in real time, making it a useful tool to evaluate the dynamics of cellular responses and to detect relevant time points when modifications occur.

Not surprisingly, the CFE was the most sensitive assay, most likely in connection to the longer exposure time and lower cell density. All the Ag NMs were classified as cytotoxic according to this test, and the EC_50_ for the Ag nanowires was lower than in AB and ECSIS. Interestingly, here the CeO_2_ stamps were cytotoxic, possibly supporting the observations on the slight reduction of proliferation/cell viability observed in the ECSIS assay for the highest concentration of this nanoform. As the ECSIS, the CFE assay is free from particles-assay interference, and thus valuable for the hazard assessment of NMs. In addition, due to the longer exposure time, the CFE could be considered as a NAM to address sub-chronic (or long term) exposures, which makes it even more interesting in view of NGRA. Although not applied here, measuring the size of the cell colonies besides their number would make the CFE assay more informative on the possible mechanisms involved in cytotoxicity, e.g. when reduced cell proliferation might be involved [53].

All data mining techniques (PCA and 2D-HCA) and machine learning technique (CT model) used in this study consistently underlined the critical role of variables such as the NMs mean size measured by TEM, the aspect ratio, the hydrodynamic diameter measured by DLS in the stock preparation, and the surface charge measured in the cell culture medium in differentiating NMs based on their cytotoxicity. This consistency across computer-aided methods highlights the robustness of these variables as key determinants for the predictive classification and grouping of NMs according to their potential hazard, based on their essential physico-chemical properties. However, it is important to acknowledge that these results may be partially limited due to the small size of the dataset, and the lack of cytotoxic effect of most of the nanoforms investigated. Notably, despite these limitations, the results clearly demonstrated a significant difference between Ag nanowires and other NMs. These were indeed the most cytotoxic nanoform, and concomitantly the one with the highest ζ-potential (T0) and the largest size and aspect ratio in the stock preparation.

Although the generalizability of these results must be thoroughly evaluated, the NMs’ aspect ratio in connection to cytotoxicity here observed is consistent with other data in the literature, as long (> 1 μm) nanofibers and high aspect ratio nanostructures (HARN) are known to have higher cytotoxicity, compared to their round counterparts [1, 59]. Therefore, this property could indeed here explain the higher cytotoxicity of the Ag nanowires.

A previous study from our group identified quantum mechanical properties, e.g., electron affinity, ionization potential, electronic energy, energy of the highest occupied molecular orbital (HOMO), energy of the lowest unoccupied molecular orbital (LUMO) and band gap energy in connection with the cytotoxicity of NMs [21]. These descriptors are related to the NMs’ solubility rate, which has been in turn correlated to toxicity [30, 39, 40]. As these properties (or descriptors) are related to the chemistry of the materials, they are constant for different nanoforms with the same chemical composition. Thus, they can explain the higher cytotoxicity of certain NMs compared to others, e.g., Ag vs TiO_2_, but not the diverse toxicity of the nanoforms of the same material.

Despite the due caution here needed, it is interesting to observe the results of the network approach similarity analysis applied. Based on the NMs’ physico-chemical properties, the model separated three nanoforms from the other materials; among these we find the most cytotoxic Ag nanowires, and the genotoxic CeO_2_ stamps. The CeO_2_ aggregates were also distanced from the other NMs, but this nanoform did not induce any effect on the biological endpoints investigated. A detailed analysis of the physico-chemical properties of these three nanoforms provides new insights into their differences from other NMs. Among the analyzed nanoparticles, Ag nanowires, CeO_2_ aggregates and CeO_2_ stamps exhibit the highest, second highest and third highest ζ-potential values in the stock preparation, respectively. In addition, Ag nanowires and CeO₂ stamps have the highest and third highest aspect ratio values, respectively, among all the NMs studied. In contrast, CeO_2_ aggregates exhibit the largest hydrodynamic diameter in cell culture medium at 0 h. These results suggest that differences in cytotoxicity and genotoxicity cannot be attributed to a single property, but rather to a combination of unique properties that must be considered together. The Ag 20 and 50 nm NPs were similar to each other in the network approach analysis and also showed similar toxicological behavior, i.e., response curves in the AB and CFE assays. TiO_2_ NPs and TiO_2_ aggregates were also closely positioned to each other, although the latter appeared to be slightly cytotoxic and with equivocal results in the comet assay, while the former was non-toxic. These results suggest that similarity analyses of nanoforms based on their physico-chemical properties might help the grouping of NMs, e.g., for read-across purposes. Further investigations are needed to explore the inclusion of a larger number of NMs and other physical parameters that were not here addressed, such as deposition efficiency, dissolution, BET (surface/volume ratio), particle surface valence, and additional biological endpoints to strengthen the approach.

## Conclusions

In this work we explored an approach to apply physico-chemical analyses and in vitro assays in combination to in silico investigations of a group of nine different nanoforms to address hazard assessment of NMs in view of NGRA and SSbD. The role of the NMs’ physico-chemical properties in eliciting biological responses was highlighted, with few nanoforms presenting specific toxicological behaviors. The importance of a proper NM characterization and of addressing nano-specific considerations when investigating and reporting toxicological data was underlined, and the application of in silico analyses in supporting hazard assessment on NMs was explored.

The small size of the dataset is clearly a shortcoming in this work and must be taken into account when interpreting and generalizing the results of the in silico analyses. Another difficulty encountered is that most of the NMs here tested were non-effective on the biological endpoints addressed, limiting the applicability of the in silico models.

Despite these limitations, all the similarity analyses (network modeling, PCA, and 2D-HCA) and SAR approach (CT model) effectively identified and separated the biologically active NMs, using only their physico-chemical properties as input. These results clearly demonstrate the strong potential of this approach to separate biologically active nanoforms from inactive ones, even when based on the same chemical element.

In future studies, the methodology could be further strengthened by applying it to a larger number of materials, incorporating more physico-chemical parameters, and utilizing additional in vitro methods and endpoints. While some findings of this study may not be fully generalizable due to the reported limitations, the approach presented provides valuable insights, effectively validating the NAMs and parameters applied, supporting their application to future studies and other materials. Previous findings on the relevance of certain physico-chemical properties, such as aspect ratio and ζ-potential, are reinforced. The potential of applying in vitro methods in perspective of NGRA is here demonstrated, cross-validating three cytotoxicity assessment methods (AB, CFE, and ECSIS), and highlighting their strength i.e. sensitivity and relevance for sub-chronic or long-term exposures (in the case of CFE), and absence of interference issues with NMs (CFE and ECSIS). As a final consideration, we would like to stress the importance to further progress with the harmonization and standardization of the SOPs for in vitro methods in general, and specifically for the use with NMs, so that data reported in the literature will be more trustable and comparable.

## Supplementary Information

Additional file 1.

## Data Availability

Data is provided within the manuscript or supplementary information files. The full datasets are available from the authors on request.
