# Supplementary material for: Hazard assessment of nanomaterials: how to meet the requirements for (next generation) risk assessment
Source: Part Fibre Toxicol. 2024 Dec 27;21:54. doi: 10.1186/s12989-024-00615-4 (PMC11674189; doi:10.1186/s12989-024-00615-4)

**Supplementary materials**

**Supplementary material 1. NMs particle size distribution by TEM imaging and UV-vis spectra.**


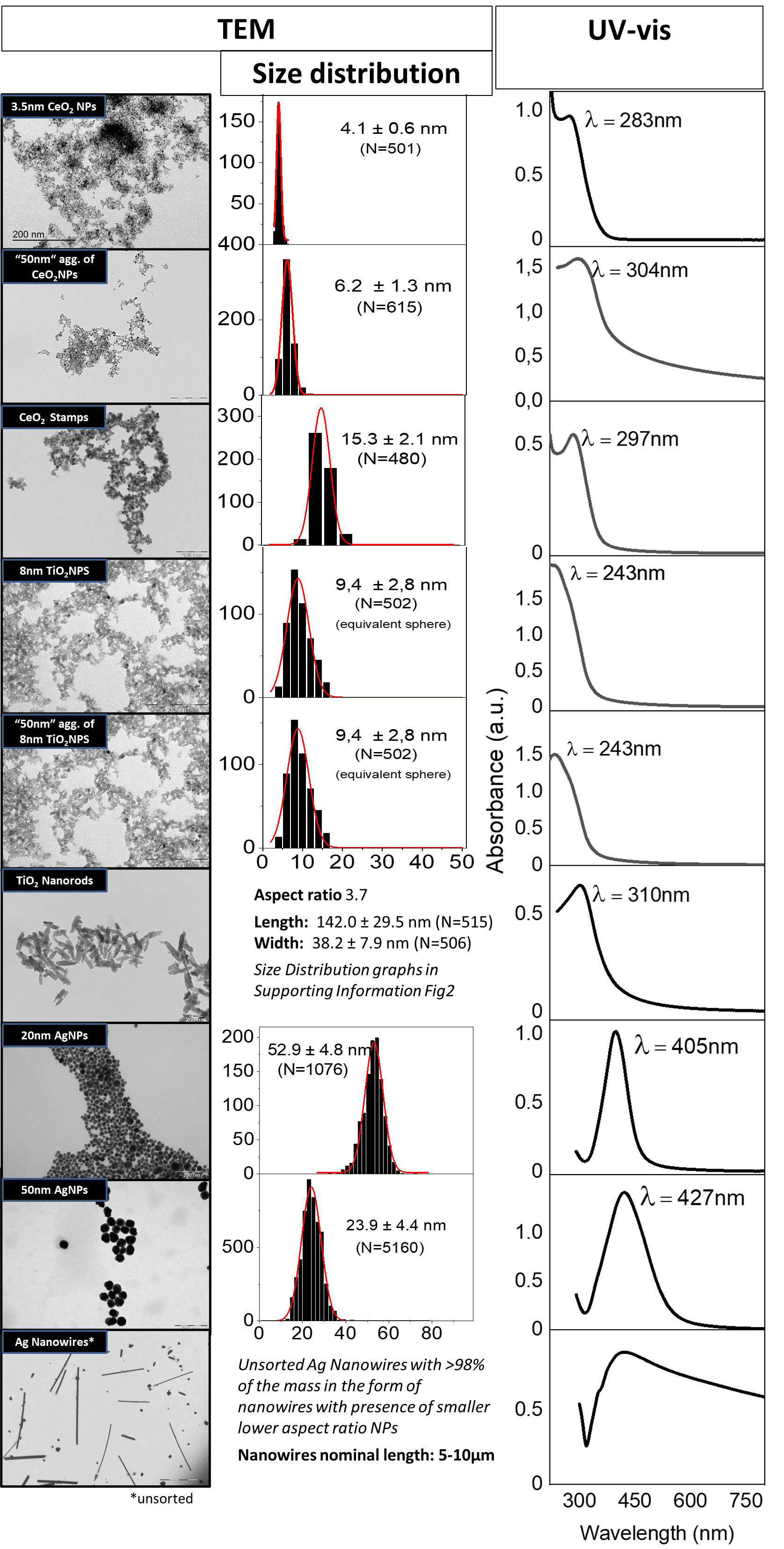


**S1 Figure 1:** NMs particle size distribution by TEM imaging and UV-vis spectra


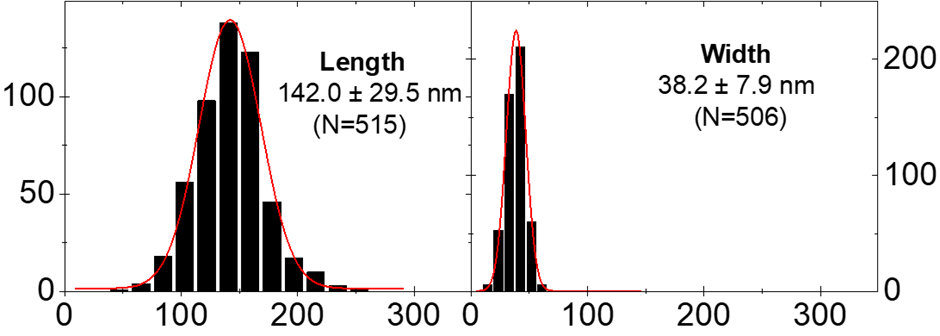


**S1 Figure 2:** TiO_2_ nanorods size distribution

**Supplementary material 2.** **X-ray Photoelectron Spectroscopy (XPS)**

The UV-visible spectrum analysis of the CeO_2_ NPs (3.5 nm) reveals a notable absorption band at 282 nm. According to the Tauc analysis, this corresponds to an optical indirect band gap energy of 3.07 eV (Fig. 1a). Similarly, the UV-visible spectrum of the CeO_2_ stamps exhibits a distinctive absorption band at 297 nm, with the Tauc analysis indicating an optical indirect band gap energy of 3.11 eV (Fig. 1c). The observed differences in absorption characteristics and band gap energies between the CeO_2_ samples may be attributed to variations in their structures, sizes and oxygen vacancies.

Further detailed analysis by XPS establishes a clear relationship with oxygen vacancies. XPS analysis was employed to determine Ce3+/Ce4+ species, recording the core level XPS spectra of Ce 3d. Through a meticulous peak-fitting process, Ce3d spectrum was analyzed using six peaks for Ce4+ (V, V″, V‴, U, U″ and U‴), corresponding to three pairs of spin-orbit doublets, and four peaks (two doublets) for Ce3+ (V0, V′, U0 and U′), based on the peak positions reported by (Mullins et al., 1998), where U and V refer to the 3d3/2 and 3d5/2 spin-orbit components, respectively. Due to the highly nonstoichiometric nature of CeO_2_, both 3+ and 4+ oxidation states coexist in the material. However, smaller 3.5 nm CeO_2_ NPs exhibit a higher concentration of oxygen vacancies (35%) than CeO_2_ stamps (23%). Relative concentrations of cerium ions were calculated from the peak areas obtaining [Ce3+] of 41% for 3.5 nm CeO_2_ NPs, 23% for the stamps and 24% for the aggregates (see the table).


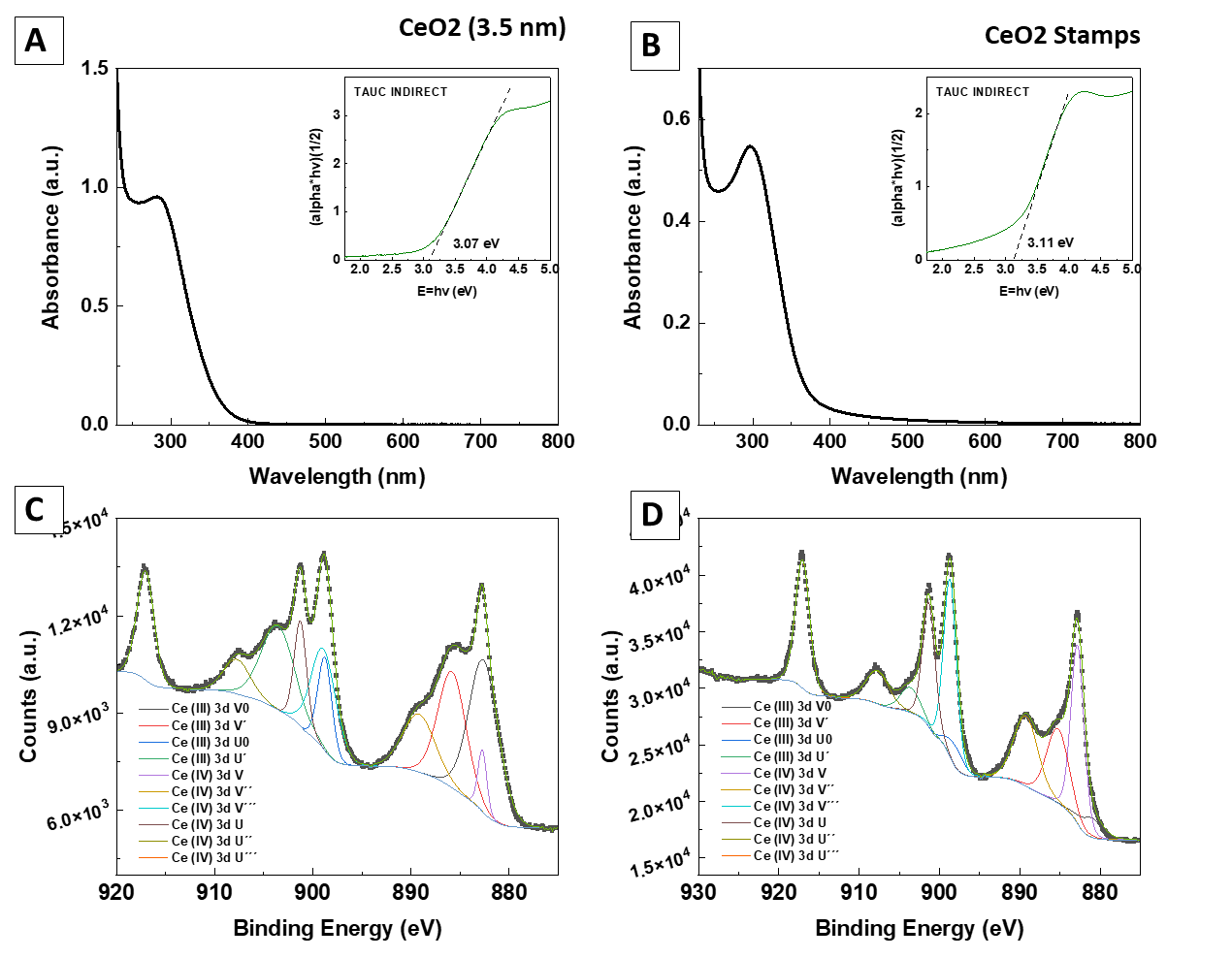


**S2 Figure 1:** Synthesis of CeO_2_ nanoform. UV-visible spectrum of the 3.5 nm CeO_2_ NPs (A) and stamps (B) and determination of the optical band gap energy by Tauc equation (inset). Ce 3d XPS spectra of CeO_2_ nanoparticles. Ce3+ peaks corresponding to v0, v’, and u0 and u’. Ce4+ peaks corresponding to v, v’’ and v’’, and u, u’’ and u’’’ components. Experimental spectra black-dotted line. Fitted spectra green-solid line.

| **Synthesis** | **% Ce(III)** | **Bandgap Energy (Indirect)** | **Urbach Energy** |
| --- | --- | --- | --- |
| **CeO_2_ NPs** | **41** | **3.07 eV** | **0.306 eV** |
| **CeO_2_ aggregates** | **24** | **3.12 eV** | **0.584 eV** |
| **CeO_2_ stamps** | **23** | **3.14 eV** | **0.571 eV** |

**Supplementary material 3. Transmission electron microscopy (TEM) imaging of cells exposed to the NMs, additional pictures.**

**
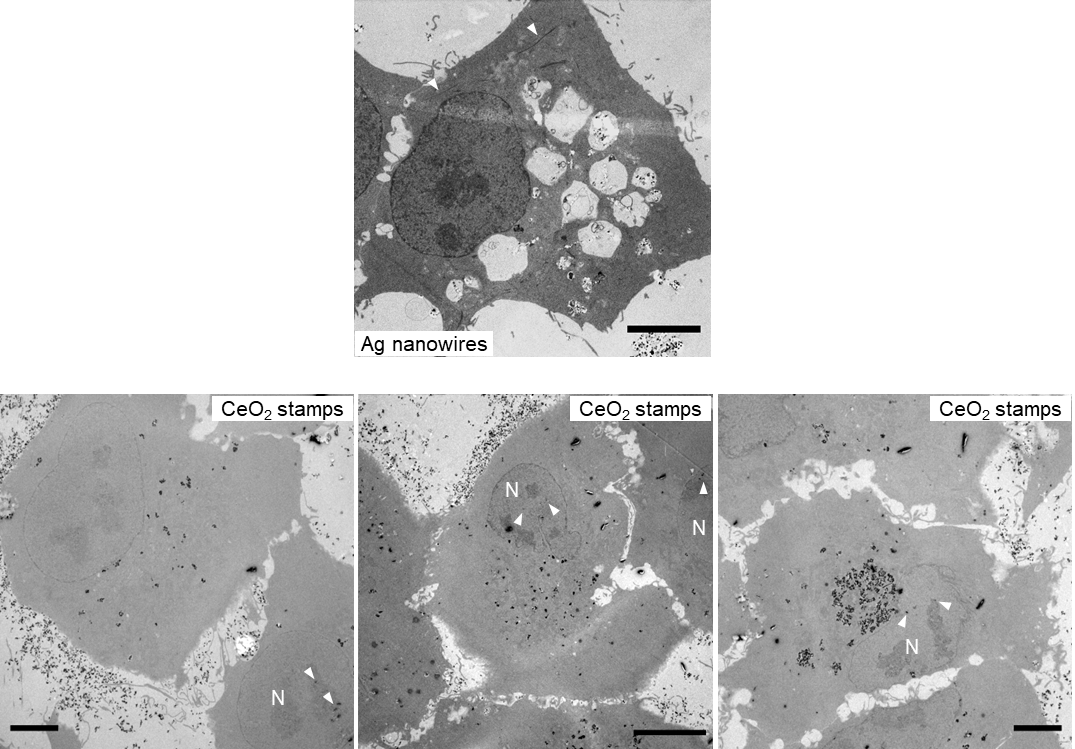
**

**S3 Figure 1:** Transmission electron micrographs of A549 cells treated for 24 h with 50 µg/ml of the Ag nanowires and CeO_2_ stamps. Arrow points to CeO_2_ stamps in the nucleus. N (nucleus). Scalebars: 5 µm in low magnifications.

**Supplementary material 4. Controls of *in vitro* assays.**

**S4 Table 1:** Alamar blue assay controls, here reported as relative fluorescence intensity (%, mean ± SD) over negative control (100 %)

|  | **3 h** | | | | **24 h** | | | |
| --- | --- | --- | --- | --- | --- | --- | --- | --- |
|  | **DC_H_** | **DC_L_** | **PC** | **Int** | **DC_H_** | **DC_L_** | **PC** | **Int** |
| **CeO_2_ stamps** | 98.4 ± 8.1 | 88.0 ± 4.6 | * 28.4 ± 10.2 | 3.3 ± 7.2 | 104.5 ± 10.8 | 95.1 ± 15.0 | * 0.1 ± 0.6 | 5.7 ± 8.1 |
| **CeO_2_ aggregates** | 114.1 ± 20.0 | 100.2 ± 6.9 | * 32.5 ± 13.0 | 0.7 ± 1.8 | 102.7 ± 9.4 | 99.7 ± 7.2 | * - 0.1 ± 0.9 | 2.4 ± NA |
| **CeO_2_ NPs** | 100.0 ± 5.6 | 95.4 ± 10.1 | * 33.0 ± 12.5 | 2.4 ± 0.7 | 103.0 ± 9.7 | 98.5 ± 13.4 | * 0.4 ± 0.8 | 2.1 ± 1.6 |
| **TiO_2_ aggregates** | 96.0 ± 9.8 | 97.8 ± 12.8 | * 34.6 ± 20.0 | -2.5 ± 7.6 | 101.0 ± 6.3 | 100.2 ± 9.6 | * 0.9 ± 2.3 | -1.6 ± 0.1 |
| **TiO_2_ nanorods** | 97.4 ± 8.9 | 105.3 ± 10.5 | * 37.2 ± 27.2 | -2.9 ± 10.9 | 98.8 ± 6.7 | 98.5 ± 12.9 | * 0.9 ± 2.1 | 1.3 ± 0.9 |
| **TiO_2_ NPs** | 95.5 ± 9.3 | 103.8 ± 10.3 | * 31.2 ± 17.7 | -7.2 ± 3.4 | 95.9 ± 7.5 | 101.3 ± 7.5 | * 0.9 ± 1.7 | -1.5 ± 1.1 |
| **Ag 50nm NPs** | 101.3 ± 4.9 | 101.3 ± 5.6 | * 15.8 ± 6.2 | 2.2 ± 0.05 | 93.4 ± 12.0 | 106.9 ± 0.9 | * - 1.0 ± 2.3 | 2.4 ± 0.5 |
| **Ag 20nm NPs** | 102.2 ± 5.7 | 105.8 ± 6.3 | * 20.2 ± 8.2 | 1.9 ± 2.6 | 100.2 ± 18.6 | 113.9 ± 5.5 | * - 0.9 ± 2.1 | 1.8 ± 2.9 |
| **Ag nanowires** | 111.9 ± 6.8 | 116.7 ± 0.04 | * 18.8 ± 7.0 | 14.9 ± 12.4 | 99.5 ± 4.2 | 117.3 ± 1.3 | * - 0.5 ± 1.2 | 5.6 ± 3.6 |

DC: dispersion medium control at the highest (DC_H_) and lowest (DC_L_) concentration used for the corresponding NM. PC: positive control, chlorpromazine (50 µM). Int: interference control (relative fluorescence intensity < 10 % indicates no interference). * Statistically significant difference respect to negative control according to one-way ANOVA.

**S4 Table 2:** CFE assay controls, here reported as relative colony forming efficiency (rCFE) (%, mean ± SD) over negative control (100 %)

|  | **rCFE(%)** | | |
| --- | --- | --- | --- |
|  | **DC_H_** | **DC_L_** | **PC** |
| **CeO_2_ stamps** | 88.1 ± 9.2 | 102.2 ± 11.7 | * 0.0 ± 0.0 |
| **CeO_2_ aggregates** | 98.5 ± 18.0 | 108.5 ± 18.4 | * 0.0 ± 0.0 |
| **CeO_2_ NPs** | 89.1 ± 16.3 | 90.5 ± 5.4 | * 0.0 ± 0.0 |
| **TiO_2_ aggregates** | 94.5 ± 3.5 | 99.0 ±15.0 | * 0.0 ± 0.0 |
| **TiO_2_ nanorods** | 106.3 ± 14.5 | 110.7 ±29.0 | * 0.0 ± 0.0 |
| **TiO_2_ NPs** | 89.6 ± 12.2 | 105.9 ± 3.9 | * 0.0 ± 0.0 |
| **Ag 50nm NPs** | 99.1 ± 12.7 | 95.2 ± 9.1 | * 0.0 ± 0.0 |
| **Ag 20nm NPs** | 95.9 ± 10.7 | 95.5 ± 4.5 | * 0.0 ± 0.0 |
| **Ag nanowires** | 101.5 ± 12.2 | 97.5 ± 8.2 | * 0.0 ± 0.0 |

DC: dispersion medium control at the highest (DC_H_) and lowest (DC_L_) concentration used for the corresponding NM. PC: positive control, chlorpromazine (30 µM). * Statistically significant difference respect to negative control according to one-way ANOVA.

**S4 Table 3:** Comet assay dispersion medium controls (DCs), here reported as DNA damage (% DNA in tail, mean ± SD)

|  | **3 h** | | | | **24 h** | | | |
| --- | --- | --- | --- | --- | --- | --- | --- | --- |
|  | **SBs** | | **net Fpg** | | **SBs** | | **net Fpg** | |
|  | **DC_H_** | **DC_L_** | **DC_H_** | **DC_L_** | **DC_H_** | **DC_L_** | **DC_H_** | **DC_L_** |
| **CeO_2_ stamps** | 5.5 ± 5.7 | 4.5 ± 2.4 | 0.03 ± 3.2 | 9.3 ± 12.4 | 6.5 ± 7.6 | 9.2 ± 8.1 | 1.7 ± 2.1 | 4.1 ± 4.4 |
| **CeO_2_ aggregates** | 8.7 ± 9.8 | 11.3 ± 14.8 | -2.1 ± 10.8 | -2.0 ± 12.4 | 4.9 ± 3.5 | 4.8 ± 3.4 | 6.8 ± 5.2 | 9.5 ± 7.8 |
| **CeO_2_ NPs** | 9.9 ± 11.3 | 9.2 ± 11.5 | -4.5 ± 10.9 | -2.4 ± 11.2 | 5.0 ± 5.0 | 4.6 ± 3.6 | 8.1 ± 6.4 | 10.3 ± 9.8 |
| **TiO_2_ aggregates** | 2.5 ± 2.8 | 2.8 ± 1.8 | -1.4 ± 2.8 | -1.5 ± 2.2 | 2.1 ± 0.7 | 2.7 ± 1.5 | -0.1 ± 0.8 | 5.1 ± 8.1 |
| **TiO_2_ nanorods** | 2.1 ± 1.3 | 2.2 ± 1.5 | -0.3 ± 1.3 | -0.4 ± 1.5 | 2.4 ± 0.2 | 4.5 ± 4.6 | 2.7 ± 4.5 | 0.8 ± 3.2 |
| **TiO_2_ NPs** | 2.6 ± 2.7 | 4.3 ± 4.5 | 1.2 ± 4.5 | 1.0 ± 4.0 | 2.8 ± 1.1 | 2.6 ± 1.6 | 1.3 ± 1.5 | 2.0 ± 1.9 |
| **Ag 50nm NPs** | 0.9 ± 0.5 | 1.7 ± 0.3 | 0.6 ± 2.2 | -0.5 ± 0.8 | 2.2 ± 1.0 | 2.3 ± 0.6 | -1.4 ± 0.8 | -0.2 ± 1.2 |
| **Ag 20nm NPs** | 1.1 ± 0.5 | 4.0 ± 2.8 | 0.1 ± 1.0 | -2.8 ± 2.8 | 1.7 ± 0.7 | 2.4 ± 0.9 | 0.3 ± 1.1 | -0.4 ± 1.0 |
| **Ag nanowires** | 0.9 ± 0.2 | 1.6 ± 0.8 | 0.9 ± 0.8 | -0.5 ± 1.3 | 3.0 ± 0.7 | 2.2 ± 0.6 | -0.8 ± 0.9 | 1.7 ± 1.5 |

DC: dispersion medium control at the highest (DC_H_) and lowest (DC_L_) concentration used for the corresponding NM; SBs: DNA strand breaks; net Fpg: net oxidative damage. * Statistically significant difference respect to negative control according to one-way ANOVA.

**S4 Table 4:** Comet assay positive control (PC) and test performance control, here reported as DNA damage (% DNA in tail, mean ± SD)

|  | **SBs** | **net Fpg** |
| --- | --- | --- |
| **PC (MMS) – 3 h** | * 21.6 ± 5.3 | * 31.5 ± 7.0 |
| **PC (MMS) – 24 h** | * 18.2 ± 4.0 | * 32.3 ± 7.5 |
| **H_2_O_2_** | * 84.1 ± 9.6 | - |
| **Ro 19-8022 + light** | * 28.4 ± 6.1 | * 34.5 ± 5.8 |

PC: positive control, methyl methanesulfonate (MMS, 200 µM); test performance controls: H_2_O_2_ (50 µM); Ro 19-8022 (2 µM) +light. * Statistically significant difference respect to negative control according to one-way ANOVA.

**S4 Table 5:** Comet assay historical negative and positive controls here reported as DNA damage (% DNA in tail, mean ± SD)

|  | **SBs** | **net Fpg** |
| --- | --- | --- |
| **NC – 3 h** | 8.3 ± 3.4 | 12.7 ± 4.0 |
| **NC – 24 h** | 7.7 ± 3.3 | 7.6 ± 6.7 |
| **H_2_O_2_** | 70.5 ± 16.8 | - |

NC: negative control; positive control (test performance control): H_2_O_2_ (50 µM).

**S4 Figure 1:** ECSIS assay’s interference controls. Impedance background signal (Cell Index) of DMEM/FBS was measured every 15 min for 24 h. Afterwards, NMs were added at 100 μg/ml (arrow) and incubated for additional 24 h. The ECSIS assay is not prone to NM interference.


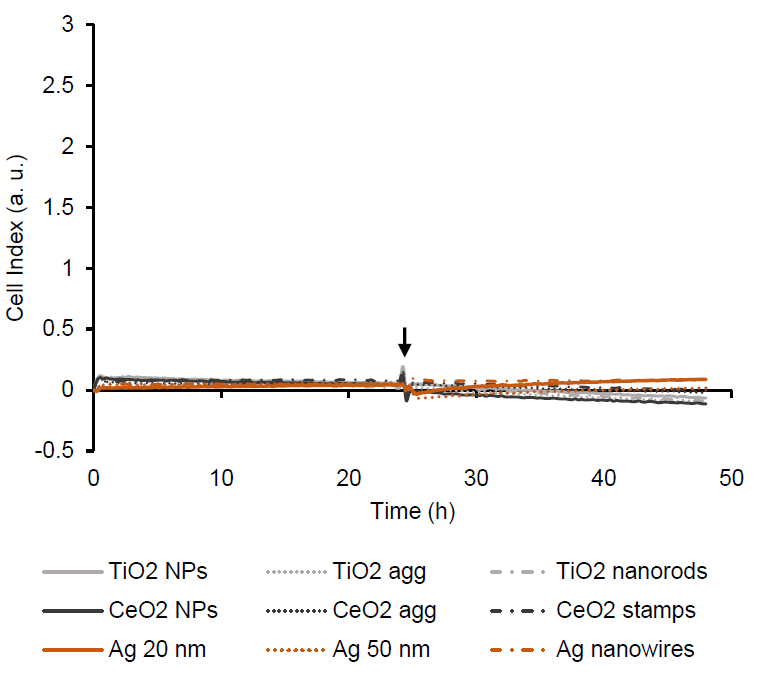


**Supplementary material 5. Representative images of CFE and Comet assay.**

**S5 Figure 1:** Representative images of the CFE assay, positive and negative controls. NC: negative control; DC: dispersion medium control; PC: positive control, chlorpromazine.
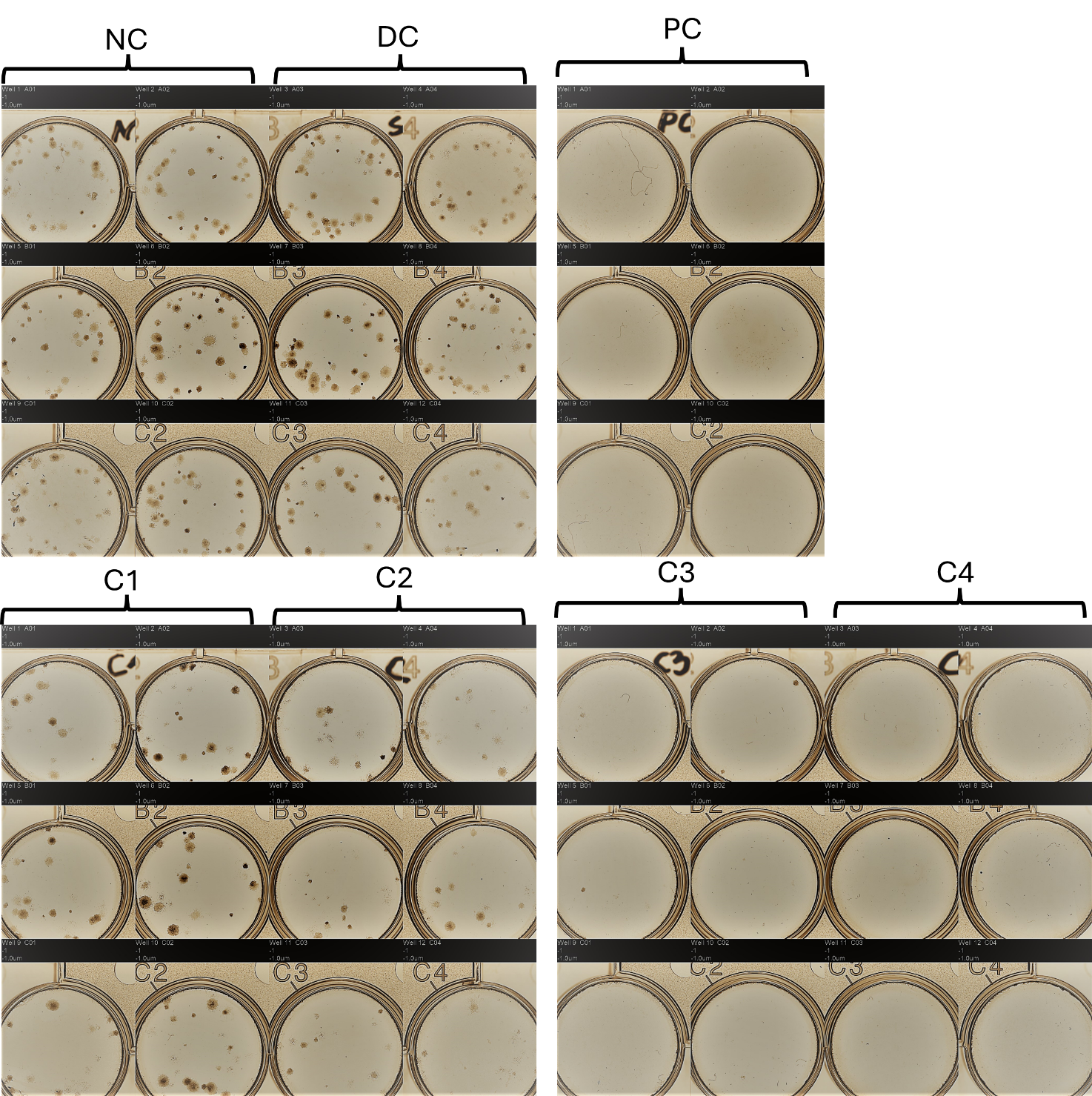


**S5 Figure 2:** Representative images of the Comet assay. Lysis: DNA strand breaks (SBs) measured by standard alkaline comet assay; Fpg: DNA oxidation lesions measured by enzyme-linked comet assay; NC: negative control; DC: dispersion medium control; PC: positive control, methyl methanesulfonate (MMS); H_2_O_2_: hydrogen peroxide, test performance control; Ro: Ro 19-8022 (2 µM) +light, test performance control. The images related to the NMs represent comets at the highest concentration tested (100 µg/ml).


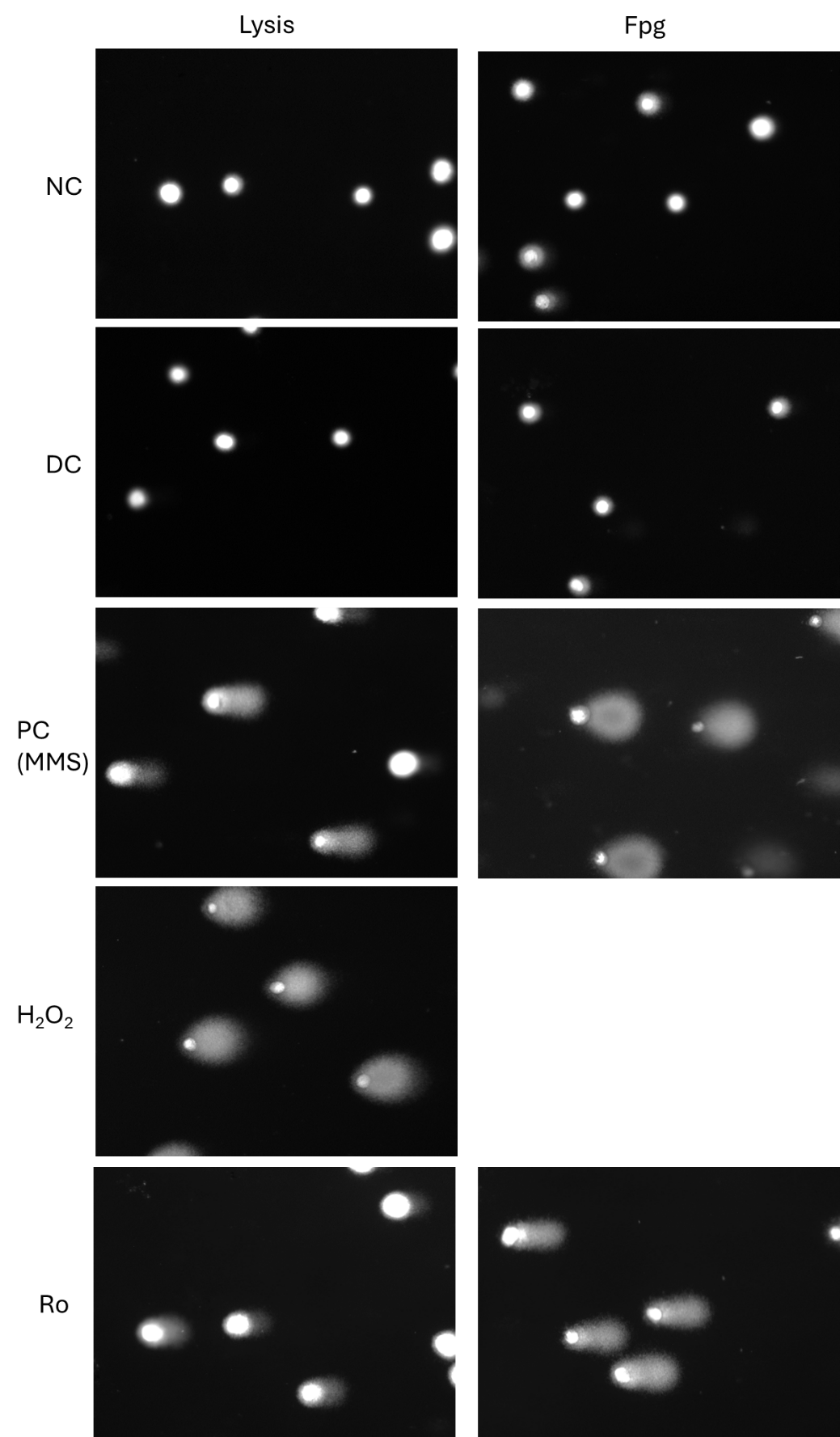


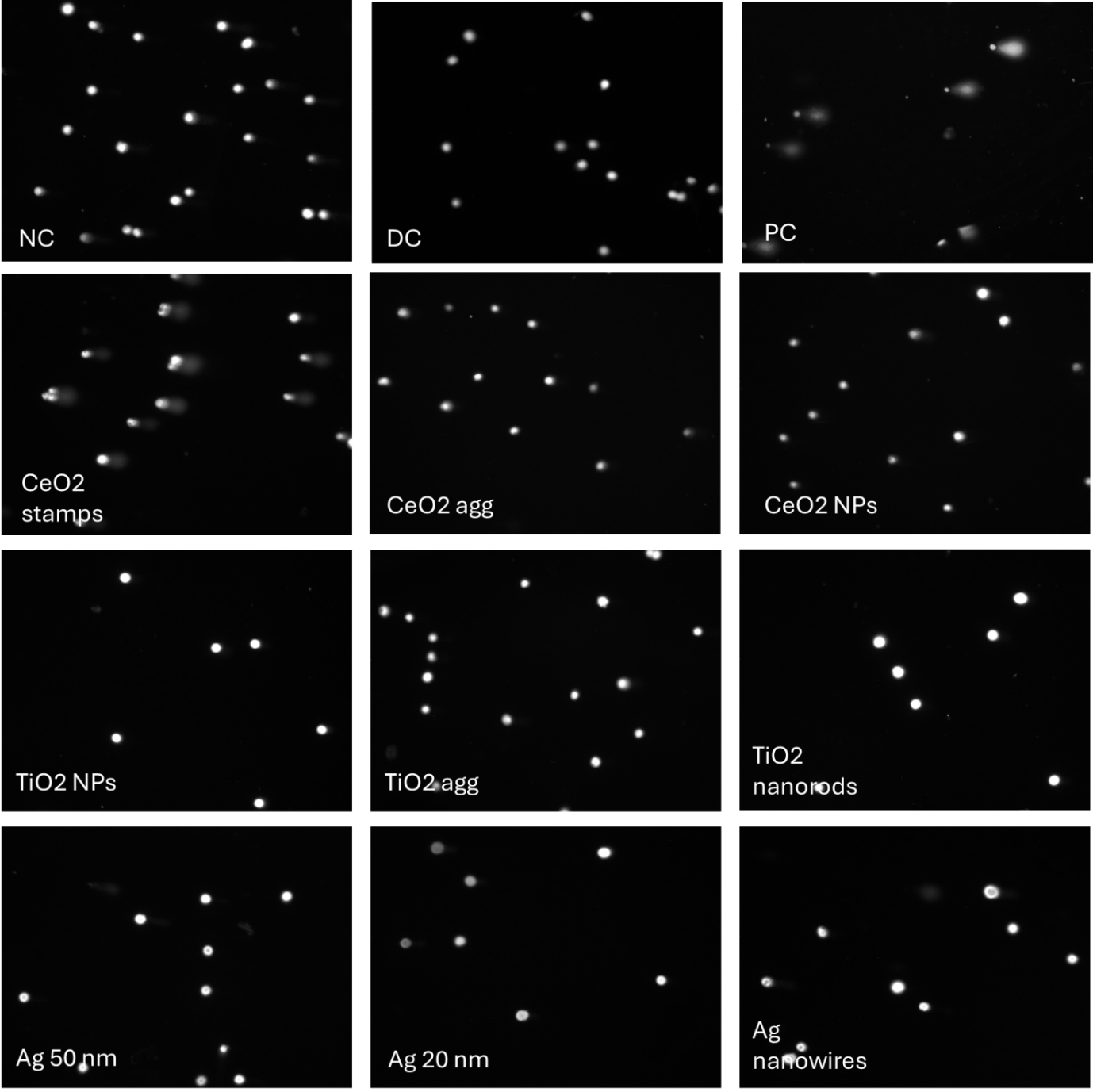

Supplement: Supplementary file 1 — Additional file 1. [file 12989_2024_615_MOESM1_ESM.docx]
